# QSSPN: dynamic simulation of molecular interaction networks describing gene regulation, signalling and whole-cell metabolism in human cells

**DOI:** 10.1093/bioinformatics/btt552

**Published:** 2013-09-23

**Authors:** Ciarán P. Fisher, Nicholas J. Plant, J. Bernadette Moore, Andrzej M. Kierzek

**Affiliations:** Faculty of Health and Medical Sciences, School of Biosciences and Medicine, University of Surrey, Guildford, Surrey GU2 7XH, UK

## Abstract

**Motivation:** Dynamic simulation of genome-scale molecular interaction networks will enable the mechanistic prediction of genotype–phenotype relationships. Despite advances in quantitative biology, full parameterization of whole-cell models is not yet possible. Simulation methods capable of using available qualitative data are required to develop dynamic whole-cell models through an iterative process of modelling and experimental validation.

**Results:** We formulate quasi-steady state Petri nets (QSSPN), a novel method integrating Petri nets and constraint-based analysis to predict the feasibility of qualitative dynamic behaviours in qualitative models of gene regulation, signalling and whole-cell metabolism. We present the first dynamic simulations including regulatory mechanisms and a genome-scale metabolic network in human cell, using bile acid homeostasis in human hepatocytes as a case study. QSSPN simulations reproduce experimentally determined qualitative dynamic behaviours and permit mechanistic analysis of genotype–phenotype relationships.

**Availability and implementation:** The model and simulation software implemented in C++ are available in supplementary material and at http://sysbio3.fhms.surrey.ac.uk/qsspn/.

**Contact:** a.kierzek@surrey.ac.uk

**Supplementary information:** Supplementary data are available at *Bioinformatics* online.

## 1 INTRODUCTION

One of the fundamental goals of molecular biology is to delineate the molecular mechanisms by which genetic information is expressed in response to environmental cues, giving rise to a specific phenotype. Given the number of molecular components of a cell and the dynamic non-linear nature of their interactions, mechanistic computational modelling is an indispensable tool. Mechanistic models formally represent the current knowledge about the molecular machinery of cells. The molecules and interactions included into such models reflect the extent of genome sequence annotation, with computer simulation used to predict system behaviour under particular environmental conditions. In systems biology, such unbiased representation of molecular interactions in mechanistic models is referred to as reconstruction (Oberhardt *et al.*, 2009). In a sense, the ultimate goal of reconstruction is to reverse engineer the entire molecular machinery of the cell in a computer model capable of reproducing the responses of specific cells/tissues to any environmental perturbation. In particular, the ability to predict the consequences of genetic background on human cell behaviour is of practical importance in clinical settings (Kolodkin *et al.*, 2012). Genetic and molecular profiling of individuals and diseased tissues is becoming increasingly important not only for the development of new therapies, but also in the design of patient-tailored treatment regimes. We believe that mechanistic prediction of genotype–phenotype relationship through computer simulation of the genome-scale molecular interaction networks in human cells is indispensable to the development of personalized medicine (Hood and Tian, 2012).

Significant effort has already been dedicated to the formal representation of existing knowledge of the molecular machinery of the cell. The predominant framework is a bipartite graph where nodes representing molecules are connected to nodes representing interactions. This representation underlies established knowledge bases such as Reactome (Croft *et al.*, 2010), BioCyc (Caspi *et al.*, 2011), KEGG (Kanehisa *et al.*, 2011) and dedicated large-scale reconstructions of cellular systems, including human cell networks relevant to major diseases (Calzone *et al.*, 2008; Oda and Kitano, 2006; Raza *et al.*, 2010). It is also reflected in major data exchange standards such as systems biology markup language (SBML) (Hucka *et al.*, 2003) and graphical notations such as (systems biology graphical notation) SBGN (Le Novere *et al.*, 2009). A major roadblock in using this wealth of formally represented knowledge to predict cellular behaviour is the lack of quantitative data about molecular amounts and interaction rates, a prerequisite to constructing dynamic models. Despite the rapid development of quantitative methods in molecular biology, full parameterization of genome-scale dynamic models, particularly of human cell systems, is still prevented by the lack of data. On the other hand, both high-throughput methods of functional genomics and traditional molecular biology routinely generate timecourse data, where dynamic changes in relative molecular activity after perturbation are monitored. Thus, there is a need to develop dynamic mechanistic computer simulation methods, capable of generating qualitative predictions about system behaviour that can be compared with available experimental data. Qualitative simulation would facilitate the iterative refinement of whole-cell mechanistic models through both theoretical prediction and experimental validation. They would also make it possible to fully use currently available network connectivity reconstructions to provide insights into the molecular mechanisms of human disease.

Although it is not yet possible to create a mechanistic model of the molecular interaction network involving all gene products, the metabolic capabilities of a whole cell can already be mechanistically simulated using constraint-based methods (CBM) (Lewis *et al.*, 2012; Price *et al.*, 2004). Briefly, a set of metabolic reaction formulas is constructed according to the complement of enzymes/transporters inferred from genome annotation. Next, a linear model of flux distribution at steady state is constructed and linear programming used to identify maximal possible flux through selected reactions in the network. This basic flux balance analysis (FBA) method can identify metabolites that can be produced given the composition of available nutrients. Even at this purely qualitative level of network description, the consequences of varying genetic background can be studied through *in silico* gene inactivation experiments. The major limitation in extending this methodology to other classes of molecular interaction is the assumption of a steady state. Owing to this assumption, essential transient behaviours such as burst or oscillations cannot be studied. However, the timescale separation between fast metabolic reactions and slow gene regulatory processes means that constraint-based models can be combined with dynamic models of regulatory processes (Covert *et al.*, 2001; 2008; Karr *et al.*, 2012; Min Lee *et al.*, 2008). These quasi-steady state methods have been used for the simulation of model microorganisms, where relatively small-scale metabolic networks have been integrated with dynamic models of regulatory processes. However, none have yet been applied to the study of the clinically relevant cellular behaviours in human tissues.

Petri net (PN) theory provides a general representation of interacting components in a network (Breitling *et al.*, 2008). PNs are bipartite graphs comprising two node classes: places and transitions. The state of the system is represented by the number of tokens assigned to each place. Transitions represent interactions within the system by moving tokens between places. This formalism naturally lends itself to the representation of molecular interaction networks, with places representing molecules, transitions molecular interactions and tokens corresponding to numbers of molecules or discrete molecular activity levels. Molecular interaction networks represented as PNs can be analyzed using a variety of simulation and formal analysis methods developed in the PN field (Rohr *et al.*, 2010). PNs have been applied in the modelling of molecular network dynamics (Breitling *et al.*, 2008; Goss and Peccoud, 1998; Mura and Csikasz-Nagy, 2008). However, of interest here is that PNs have also been shown to be advantageous for qualitative modelling of gene regulatory networks (Grunwald *et al.*, 2008; Steggles *et al.*, 2007) and signalling pathways (Ruths *et al.*, 2008a, b), where network connectivity is analyzed without knowledge of quantitative molecular concentrations and interaction rate constants.

Here, we describe quasi-steady state PNs (QSSPN), a novel algorithm capable of qualitative dynamic simulation of genome-scale molecular interaction networks including all classes of molecular interactions. We apply the algorithm to simulation of the interplay between whole-cell metabolism and gene regulatory and signalling networks involved in bile acid (BA) homeostasis in human hepatocytes. To our knowledge, this is the first dynamic simulation of a molecular interaction network involving gene regulation, signalling and full genome-scale metabolic network (GSMN) in a human cell. The unique feature that enables QSSPN to perform this simulation is Monte Carlo sampling of the feasible dynamic sequences of molecular transitions in the qualitative quasi-steady state model. In the following sections, we introduce QSSPN method, demonstrate its application to human hepatocyte simulation and compare its features with other approaches.

## 2 METHODS

### 2.1 QSSPN algorithm

**Fig. 1.**
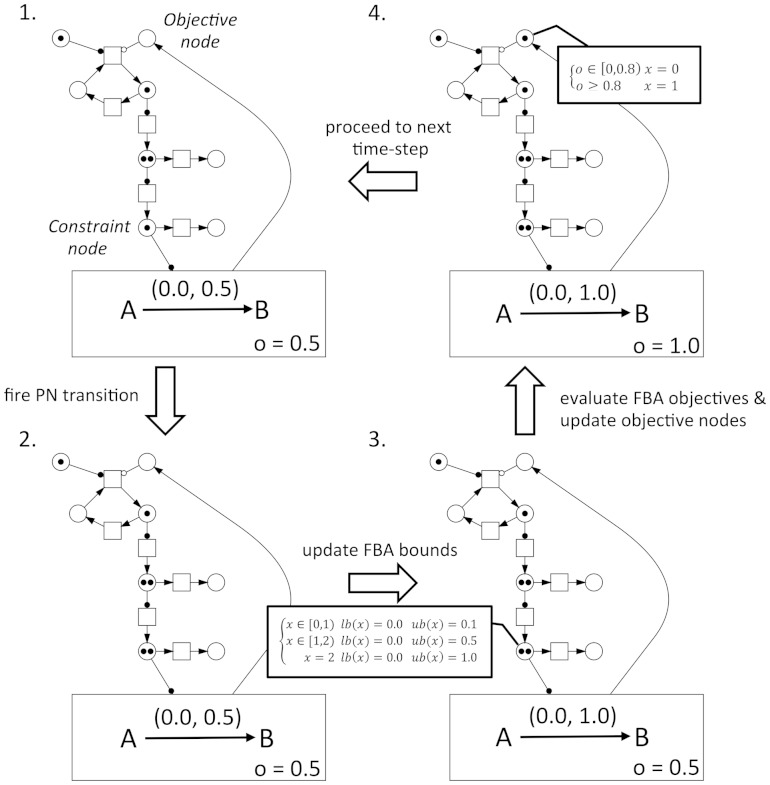
Overview of quasi-steady state PN simulation. The DT component of the model is represented using the PN formalism. Circles denote places; dots within circles are tokens; squares are transitions; arrows are edges; open circle headed arrows are inhibitory edges; and full circle headed arrows are read edges. Constraint and objective nodes couple dynamic PN to QSSF network leading to synthesis of metabolite B. The constraint node sets bounds of the rate limiting reaction producing B. The objective node requests evaluation of the maximal flux towards metabolite B by FBA. The objective value (o) at initial state is 0.5. The four basic stages of a single QSSPN iteration are shown: (**1**) firing of PN transition leads to increased number of tokens on constraint node. The pre-place and transition that fired are connected by a read edge, hence no token was consumed. (**2**) The metabolic flux bounds are updated using the lookup table associated with that constraint node. (**3**) FBA is executed to evaluate the objective function and the associated lookup table, mapping objective function value to the number of tokens, is used to update the state of the objective node. (**4**) The algorithm proceeds to next iteration. Multiple iterations of steps 1–4 generate a single token game trajectory

Figure 1 shows an overview of the QSSPN method. The molecular interactions are divided into two sets: quasi-steady state fluxes (QSSF), representing metabolic reactions; and dynamic transitions (DT), representing other classes of molecular interaction, such as signalling pathways and gene regulation. QSSF are interrogated through CBMs, whereas DTs are represented using extended PN formalism (Breitling *et al.*, 2008).

The activities of molecular species represented by PN places are given by an integer number of tokens. In most instances, tokens represent discrete levels of activity, but fully quantitative description is, in principle, possible, with node token status reflecting molecular quantities. QSSF and DT interaction sets are connected by two classes of PN places: constraint places set flux bounds in QSSF, translating place token status in to flux bounds; objective places represent metabolic outputs of the QSSF network.

Each molecular interaction in a PN subset is represented by a transition connected to pre-places representing substrates, activators and inhibitors and post-places representing products. Substrates and products are connected to transitions by standard edges, whereas catalytic activators and inhibitors are connected to transitions by read and inhibitor edges, respectively. When a transition fires, tokens from substrate pre-places are consumed and tokens representing products fired to post-places; the states of pre-places connected to transitions by read or inhibitor edges are not changed. In the simplest definition of system dynamics, transitions can fire if each substrate and activator pre-place has at least one token and all inhibitory pre-places have no tokens. To allow formulation of general rules involving token number thresholds, we define transition propensity Equation (1):
(1)
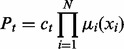

where P_t_ is a propensity function of transition t, c_t_ is a rate constant, N is the number of pre-places of transition t, x_i_ is the number of tokens at pre-place i and μ_i_ is the pre-place activity in the transition depending on the pre-place state. The activity function is a look-up table of T thresholds t_i_ and activities a_i_ allowing general definition of the pre-place contribution to the transition propensity Equation (2):
(2)
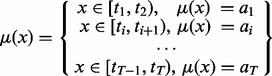



The interpretation of a transition propensity during simulations is dependent on the transition class: stochastic, continuous or immediate. Stochastic transition propensity is interpreted as the probability density of the transition firing in the next, infinitesimally small, time step. Continuous transitions are updated after each iteration of the simulation, and the number of tokens moved is proportional to the propensity (i.e. the propensity is used as reaction rate). Immediate transition fires once whenever its propensity function is different than 0. To further enhance the power of our method to integrate experimental data, we allow stochastic transitions to be delayed (Bratsun *et al.*, 2005).

Supplementary Figure S1.1 shows the algorithm that is used to generate a single dynamic trajectory of the QSSPN network, which following PN nomenclature will be referred to as a token game trajectory. The algorithm is based on the Gillespie algorithm (Gillespie, 1977) and the hybrid maximal timestep method (Puchałka and Kierzek, 2004), making it numerically stable and facilitating interrogation of modular models reconstructed at varying levels of detail. Detailed formulation of the QSSPN algorithm is provided in Section 1 of the Supplementary Materials.

### 2.2 Monte Carlo sampling of token game trajectories

A major feature of QSSPN simulations is the Monte Carlo sampling of token game trajectories. Multiple simulations, starting from the same initial conditions, output a sample of token game trajectories differing as the result of alternative sequences of stochastic transitions. Trajectories are examined to detect occurrences of dynamic behaviours of interest. Occurrence of a single trajectory exhibiting the behaviour of interest indicates that there exists a sequence of interactions, permissible with the given network connectivity, that mechanistically explains the behaviour. The major application of token game trajectory sampling is then to predict effects of system perturbations, such as gene inactivation or inhibition of molecular interaction. The number of trajectories exhibiting behaviour of interest is calculated before and after perturbation. The binomial probability confidence intervals are then used to determine whether perturbation significantly affects the fraction of trajectories exhibiting the behaviour. The simulations of human hepatocyte presented in this work demonstrate that comparative token game trajectory sampling provides an invaluable tool for mechanistic prediction of the effects of genetic polymorphisms and environmental changes on dynamic cellular behaviour. It enables qualitative simulation where tokens are interpreted as discrete activity levels of molecular species. The maximum numbers of tokens allowed on the network nodes are low and only a few discrete activity levels are considered. The rates are all set to the same value, meaning all stochastic transitions, for which input conditions are satisfied, are equally likely to happen; time is interpreted qualitatively, with the time axis reflecting the order of events rather than actual time. Our results show that this approach is successful in using qualitative reconstructions of molecular interaction networks to predict results of qualitative molecular biology experiments. Finally, we would like to note that formulation of our algorithm allows incorporation of rate constants as they become available. In a fully quantitative scenario, the number of tokens can represent the number of molecules in the cell and equations 1 and 2 can express mass action kinetics with rate constants. Quasi-steady state simulation would then produce molecular number timecourses in real time.

## 3 RESULTS

### 3.1 General model of gene expression

**Fig. 2.**
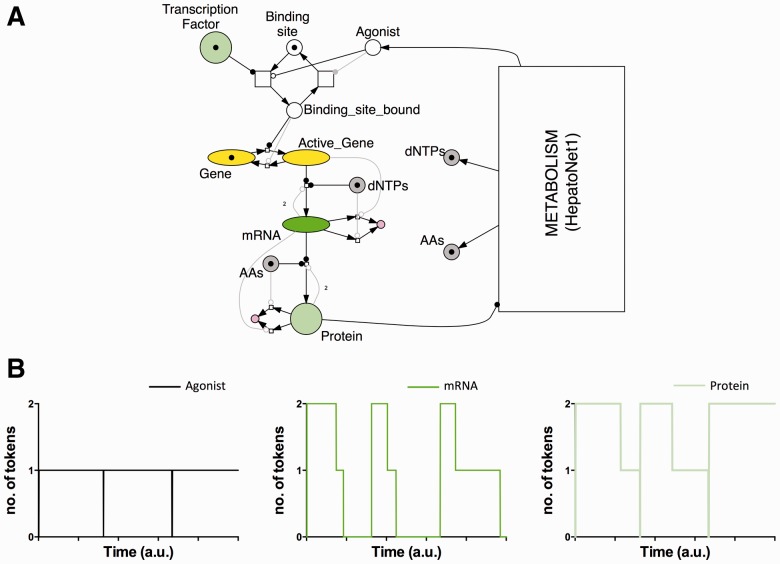
Qualitative gene expression model. (**A**) QSSPN network diagram using graphical notation based on SBGN is used to distinguish PN nodes representing DNA elements (yellow oval), RNA molecules (green oval), proteins (large green circles) and degradation products (small pink circles). Light grey colour is used for edges that are used solely to express rules rather than physical interactions. Read and inhibitory edges are headed by full and open circles, respectively. (**B**) Single token game trajectories. In qualitative QSSPN simulation, molecular activities are represented by 0, 1 or 2 tokens and a time axis is used to represent order of events rather than physical time. Transcription initiation conditions are satisfied at the initial state. Subsequently, transcript and protein levels reach maximal level. Enzymatic activity of the protein enables synthesis of agonist, which shuts down transcription. The mRNA degradation is then followed by protein degradation. In the absence of enzyme, agonist is not made, which enables transcription. Importantly, the qualitative trajectory exhibits oscillatory dynamic behaviour of negative feedback

Regulation of gene expression is a key cellular process that needs to be included in any attempt to mechanistically simulate genotype–phenotype relationship. To enable QSSPN simulation of molecular networks in human cells, we have constructed a general model of gene expression, using qualitative rules to describe the relationship between gene regulation, transcription, translation, precursor availability and messenger RNA (mRNA)/protein degradation (Fig. 2). PN formalism allows us to incorporate established SBGN and also use graphical notation to represent model rules.

The gene expression model is illustrated using a hypothetical gene operating in a human hepatocyte. The ‘Gene’ place is used to represent the presence or absence of the gene in the genome, represented as 0 or 1 token, respectively. The ‘Active_Gene’ place represents the presence of a fully assembled transcription initiation complex for the respective gene, meeting the conditions for transcription. This is modelled by the transition that requires ‘Active_Gene’ to fire and places tokens on the ‘mRNA’ place. The PN read edge is used to ensure that the transition does not consume tokens from ‘Active_Gene’. Translation is modelled in a similar way; it requires the presence of ‘mRNA’ and places tokens on ‘Protein’ place without consuming ‘mRNA’. Degradation transitions remove tokens from both ‘Protein’ and ‘mRNA’ places. The flexibility of the PN formalism is used to express dependency between synthesis and degradation as a set of rules. Transitions and edges exclusively describing model rules are shown in grey to distinguish them from PN elements representing biological processes. The inhibitory edge that connects ‘Active_Gene’ with ‘mRNA’ degradation introduces the rule that there is no net mRNA degradation if transcription is possible. We then introduce a second rule that prevents the unnecessary firing of the transcription reaction, when ‘mRNA’ has two tokens, which is a maximal number of tokens in this model. This rule is implemented as an inhibitory edge, which sets propensity of transcription to 0, if ‘mRNA’ has two tokens. Together, these rules result in ‘mRNA’ place acquiring two tokens when conditions of transcription are satisfied and remaining at this state as long as the transcriptional pre-conditions are met. When conditions for transcription are no longer satisfied, the degradation transition removes tokens from the ‘mRNA’ place. Translation and protein degradation are modelled in the same fashion. Thus, we can describe the rise of a transcript/protein to two functional levels and its subsequent degradation in a rule-based model that uses only a few discrete activity levels and is computationally efficient.

Our PN model of gene expression also includes the metabolic demands of transcription and translation processes. Although the model shown in Figure 2 is for method illustration purposes only and does not represent any existing biological system, we have coupled the gene expression model to a published GSMN reconstruction of human hepatocyte whole-cell metabolism (HepatoNet1) (Gille *et al.*, 2010). This allows us to test integration of the gene expression model with a realistic scale model of metabolic demands. The ‘dNTPs’ and ‘AAs’ objective nodes test the sum of fluxes towards the 4 nucleotides and 20 amino acids, respectively. In a qualitative model only the producibility of all precursors is required, with both objective nodes assuming the state of 1 if the objective values are more than a threshold of 10^−^^3^ (equivalent to testing ‘greater than 0’ condition). The ‘dNTPs’ and ‘AAs’ are connected to transcription and translation transitions as logical pre-places, modelling the precursor requirement of these processes. Logical PN nodes (depicted as shaded places) are an established feature of extended PN formalism, meaning that every ‘dNTPs’ and ‘AAs’ node has the same token status across a PN network. An additional rule is introduced to trigger net degradation of mRNA/protein if transcription/translation stops because of a lack of precursors.

To complete our illustration of a general gene expression model and its coupling with whole-cell metabolism, we have introduced transcriptional regulation by a transcription factor that senses availability of a small molecular agonist produced by metabolic network and made synthesis of this metabolite dependent on enzymatic activity of a protein produced by the ‘Gene’. We use intracellular cholate as an agonist and cholesterol-7-alpha-monooxygenase (CYP7A1) for enzymatic activity. The agonist is an objective node, the state of which depends on maximal producibility of intracellular cholate. The ‘Protein’ is a constraint node, which sets bounds of CYP7A1 catalyzed reactions in HepatoNet1. We then model interference of the agonist with transcription factor binding at the site controlling transcription initiation. The free and bound state of the binding site is represented by two PN places, with transcription factor and agonist affecting transitions between both forms (i.e. the binding site is bound if transcription factor is present and agonist is absent).

We used the model to perform qualitative simulation of gene expression dynamics (Fig. 2B), with the PN node state of two tokens being used to represent a steady state where transcript and protein are functional. Time has been used to order the sequence of events rather than to represent physical time, and is expressed in arbitrary units (a.u.) and relative rates in 1/a.u. We assume that formation of a transcriptionally active gene complex is rate limiting and use immediate transcription and translation transitions. We use stochastic transitions, with a relative rate of 100/a.u. for the formation of transcriptionally active complex, so in the case of a realistic system with multiple genes, the genes are fired asynchronously and multiple trajectories with different sequences of gene activation are sampled. The dissociation of stable complex between transcription factor and binding site was assumed to be slow and was modelled by a stochastic transition with a relative rate of 10/a.u. Stochastic transitions with the rates of 1/a.u. were used for degradation reactions. All other processes were assumed to be fast and were modelled by immediate transitions. We would like to stress that rates and activity thresholds are used to express qualitative rules.

The timecourses of gene expression dynamics qualitatively reproduce the major qualitative features of gene expression behaviour and its coupling with whole-cell metabolism. When transcription activation conditions are satisfied, transcript and protein quickly reach their functional steady states. When enzymatic activity of the protein enables synthesis of the agonist, transcription is no longer possible, mRNA and protein degradation processes are no longer balanced by synthesis and both transcript and protein products degrade. This negative feedback leads to oscillations and demonstrates that QSSPN can generate qualitative trajectories exhibiting complex dynamic behaviours. In the following case study, we will demonstrate that these trajectories are directly comparable with readily available experimental data. Moreover, the simulation of these trajectories is computationally efficient and crucial for generation of trajectory samples (section 8 of Supplementary Materials). The oscillatory behaviour can be modelled with just two activity levels and thus the number of molecular events that need to be simulated is relatively small. This is important, as any change of state may potentially trigger evaluation of objective functions of whole-cell metabolic model, the most computationally expensive step of the QSSPN. Thus, apart from addressing the major challenge of sparse quantitative information, the rule-based approach shown here has a substantial computational advantage over ordinary differential equation (ODE) or stochastic simulation; in quantitative models many state changes would have to be simulated to model the balance between synthesis and degradation, requiring a lot of CBM evaluations. In contrast, our novel rule-based gene expression model generates complex qualitative dynamics with far fewer state updates.

### 3.2 Dynamic simulation of BA homeostasis

To demonstrate the power of our approach, we applied QSSPN to the complex system of BA homeostasis in human hepatocytes, the major cell type within the liver. Primary BAs are produced by hepatocytes as the products of cholesterol catabolism. They are vital for the intestinal absorption of lipophilic nutrients and the hepatobiliary secretion of endogenous and xenobiotic metabolites. They also act as signalling molecules and play important roles in the regulation of glucose lipid metabolism in the enterohepatic system and peripheral tissues (Chiang, 2009; Li and Chiang, 2012). Given their essentiality for health, but toxicity at high levels, it is not surprising that BA synthesis is tightly regulated. Despite the importance of BA homeostasis, there are limited quantitative measurements of molecule numbers and reaction rates of this system, necessitating qualitative modelling. Thus, our test case represents a typical situation faced by modellers; detailed quantitative measurements are in general not available for clinically relevant molecular interactions networks. In the Discussion and Supplementary Materials, we summarize evidence that QSSPN is a method of choice for qualitative modelling of this system.

Using the QSSPN framework, we have reconstructed a molecular interaction network representing whole-cell metabolism of hepatocyte coupled to the gene regulation and signalling processes known to be involved in BA homeostasis. For the QSSF, we use the published HepatoNet1 whole-cell reconstruction of human hepatocyte metabolism (Gille *et al.*, 2010). The objective nodes in our reconstruction monitor producibility of the BAs chenodiol and cholate in the cytoplasm, endoplasmic reticulum, bile canalicular and sinusoidal space; the producibility of cholesterol was also monitored in these compartments, allowing simulation of regulatory feedback. Intracellular BAs are farnesoid X receptor (FXR) agonists, regulating transcription through the FXR response element, inverted repeat (IR)1. FXR functions as a heterodimer with retinoid X receptor (RXR)α, although the exact molecular events of this interaction are unknown (Chiang, 2009; Kalaany and Mangelsdorf, 2006). As such, we have defined qualitative rules such that transcriptional regulation of genes through IR1 requires the presence of both FXR and RXRα proteins. Transcriptional regulation through the liver X receptor (LXR) response element direct repeat (DR)4 was modelled in the same way, requiring the producilibility of endogenous LXR agonist, hydroxycholesterol, another cholesterol catabolite and the presence of both LXRα and RXRα (Kalaany and Mangelsdorf, 2006; Zhao and Dahlman-Wright, 2010). DR1 requires the binding of both fetoprotein transcription factor (FTF) and hepatocyte nuclear factor (HNF)4α for transcriptional activation of its regulated genes. In contrast, small heterodimer partner (SHP) and cFOS individually inhibit transcription of DR1 regulated genes. Nucleotide and amino acid producibilities were included as metabolic feedbacks required for gene expression, as described previously. Diacylglycerol (DAG) and cyclic adenosine monophosphate (cAMP) producibilities were also included as metabolic inputs into the mitogen-activated protein kinase (MAPK) signalling pathway. Qualitative rules were constructed to reproduce a homeostatic response of the system to an increase in intracellular cholesterol within maximal simulation time. The full set of rules and a network diagram of the hepatocyte model are given in the supplementary materials (Supplementary Table S3.1 and Supplementary Fig. S2.1).

**Fig. 3.**
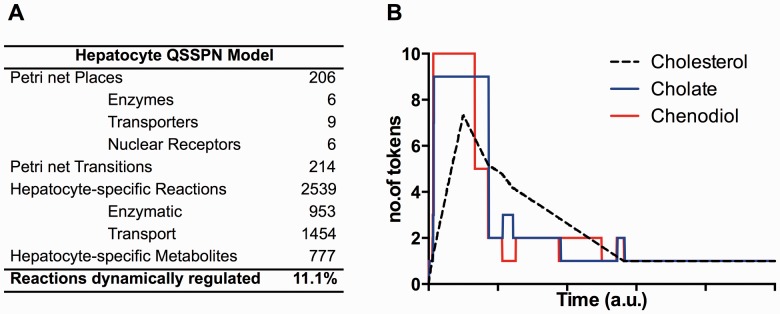
QSSPN model of the global metabolism regulation maintaining BA homeostasis in human hepatocyte. (**A**) Summary table of hepatic model composition. (**B**) Example, single QSSPN trajectory showing homeostatic response to cholesterol perturbation. The continuous piecewise-linear cholesterol perturbation is modelled by continuous transitions and large numbers of tokens. Token numbers in the cholesterol timecourse have been divided by 100 000 to fit onto the plot. The synthesis flux for both BAs quickly rises to maximal level in response to increasing cholesterol availability. Subsequently, the regulatory network decreases the BA flux to basal level

Figure 3 presents statistics of our reconstruction and an example qualitative trajectory exhibiting a homeostatic response. Rising availability of intracellular cholesterol results in the increase of BA fluxes to maximal levels; cholesterol is cleared through conversion to BAs plus increased transport reactions. When the cholesterol source is depleted, the level of intracellular BAs starts to decrease. During this time the regulatory network responds to the high BA flux by down-regulating conversion of cholesterol to BAs. BA flux is stabilized at the basal level, slowing the rate of cholesterol clearance, which is then dependent on efflux transport alone. In summary, the system responds to the burst in cholesterol availability by altering network fluxes to maximize cholesterol clearance, but prevents prolonged synthesis of large quantities of BA, mitigating against their potential toxicity. Simulated trajectories demonstrate that a dynamic response reproducing known biology is feasible in this hepatocyte model. It is worth noting that modelling the activity of only six nuclear receptors, controlling 15 genes, the network regulates the activity of 269/2539 metabolic reactions. This highlights the importance of modelling dynamic regulatory networks to facilitate the study of mechanism, crosstalk and feedback.

**Fig. 4.**
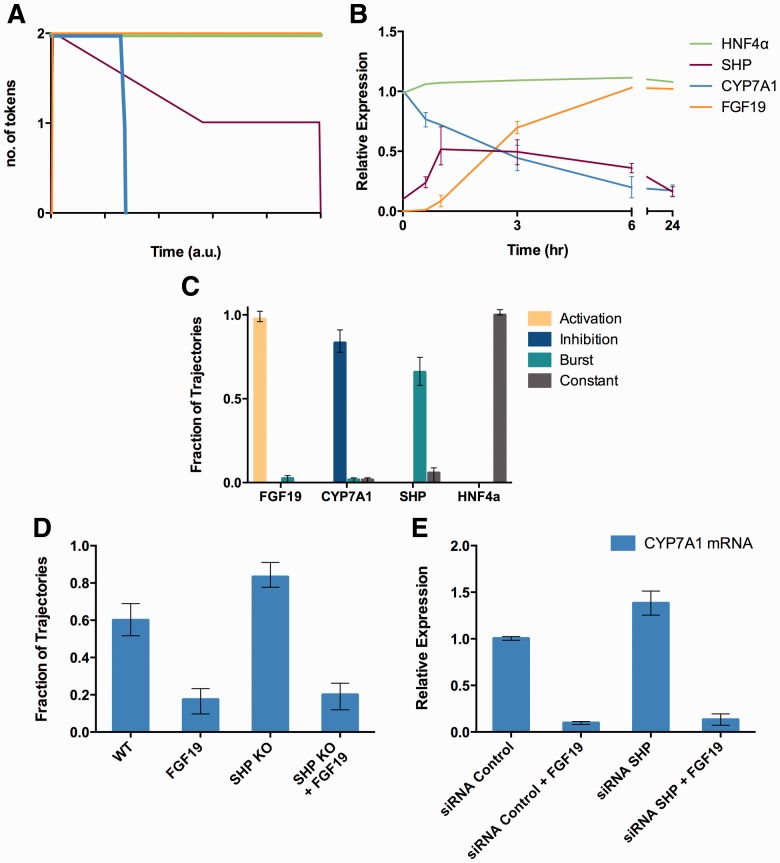
QSSPN simulations with hepatocyte model reproduce experimental data. (**A**) Example trajectory of the simulation of relative transcript level timecourses, reproducing (**B**) experimental data from treatment of primary human hepatocytes with FXR agonist, GW4064. In agreement with experimental data, CYP7A1 transcription is inhibited, FGF19 mRNA level increases, SHP mRNA transcription is activated followed by inhibition (burst) and HNF4α remains constant. (**C**) The fraction of trajectories in which CYP7A1, FGF19, SHP and HNF4α transcripts exhibit activation, inhibition, constant and burst behaviours in response to FXR-specific agonist, GW4064. (**D**) The fraction of trajectories in which CYP7A1 transcript levels increase in the original unperturbed model (WT), with permanent FGF19 expression, with SHP siRNA and the subjected to both SHP siRNA and FGF19 perturbations. Results are in agreement with experimental data (**E**). C and D show results of 120 trajectories, error bars show 95% binomial probability confidence intervals. B and E show experimental results digitized from Song *et al.* (2008). Relative expression data in B were transformed to fit onto one plot, as such the plot should only be interpreted in terms of qualitative behaviours

Quantitative measurements of molecular amount timecourses are not available for this system, but qualitative experimental data on responses to perturbations are a common situation in the literature. A powerful feature of QSSPN is its capacity to make use of qualitative data to validate and refine qualitative models. Using our QSSPN hepatocyte model, we simulated the published experimental conditions of Song *et al.* where cultured human hepatocytes were treated with an FXR-specific agonist and subject to small interfering RNA (siRNA) knock-out of SHP (Song *et al.*, 2008). Simulations were started from an initial state where there was one token on each gene place and all other PN nodes token states 0, representing the genotype of the system. Simulations were run for one arbitrary time unit to allow all genes to reach basal expression levels, and the FXR permanently activated to simulate treatment with the FXR-specific agonist, GW4064. We compared the results of 120 simulated trajectories, with relative mRNA expression data obtained by qRT-PCR in the original publication (Fig. 4). In Section 4.1 of the Supplementary Material, we present detailed definitions of qualitative behaviours, which are used to evaluate both experimental data and simulation results, thus ensuring direct comparison of simulation and experiment.

The experimental data (Fig. 4B) show that following GW4064 treatment, CYP7A1 transcription is inhibited, SHP shows a burst in expression, FGF19 expression is activated and HNF4α transcripts remain constant. Despite a small sample size we can establish that each of the transcripts exhibits experimentally determined behaviours with a probability higher than upper 95% confidence interval of other behaviours. To robustly simulate experimental data, it was necessary to incorporate the negative auto-regulation of the SHP gene, as described by Chanda *et al.*(Chanda *et al.*, 2010). Without this auto-regulation, SHP exhibits the same prolonged activation as FGF19 rather than a burst behaviour. Moreover, our preliminary simulations indicated that to reproduce the single burst behaviour of SHP, as reported by Song *et al.*, SHP protein must be set as stable. The necessity of this stability was supported by experimental data, where SHP stability increased at least 6-fold in the presence of BA (Miao *et al.*, 2009). Thus, adjustment of the qualitative parameters required to reproduce experimental data of Song *et al.* agrees with published experimental data (Miao *et al.*, 2009) that was not used to refine the model. This demonstrates that qualitative modelling in QSSPN framework is capable of providing novel mechanistic insight. Next, we evaluated performance of our model in predicting the effects of siRNA knock-outs in the response to recombinant FGF19 treatment. We calculated the fraction of trajectories in which CYP7A1 mRNA transcript levels reached maximum (two tokens) and remained at maximum until the end of each simulation. The system was then perturbed to mirror either exposure to exogenous FGF19 and/or SHP knock-down via siRNA. Setting the FGF19 protein node to the maximum level of two tokens and removing protein degradation transitions simulated treatment with FGF19. Knock-down of SHP via siRNA was simulated by introducing an ‘NR0B2_siRNA’ node connected by an inhibitory edge to the SHP ‘translation’ transition node and connected by a read edge to an additional SHP mRNA degradation transition with a high rate of 1000, abolishing SHP gene expression at the mRNA level. Again, simulation results reproduced the qualitative observations of experimental system behaviour (Song *et al.*, 2008); treatment with FGF19 resulted in a decrease in CYP7A1 activation, and FGF19 inhibition of CYP7A1 transcript did not change as the result siRNA transfection. Moreover, QSSPN reproduced the increase in CYP7A1 expression after siRNA transfection. The Supplementary Material shows (Section 4) evaluation of QSSPN predictive power against the complete set of experimental data available in Song *et al.* (2008). The method achieves high correlation with experimental data (Matthews correlation coefficient of 0.812) and only fails to predict 2/14 experimental behaviours available in the original experimental publication. We also demonstrate that QSSPN parameters are qualitative, perturbation of their quantitative values does not affect results (Supplementary Material Section 5). Finally, we argue that further refinement of the model, to reproduce all 14 experimental behaviours available in Song *et al.* (2008) would require quantitative fine-tuning of parameters and would result in over fitting.

### 3.3 Analysis of genotype–phenotype relationship

**Fig. 5.**
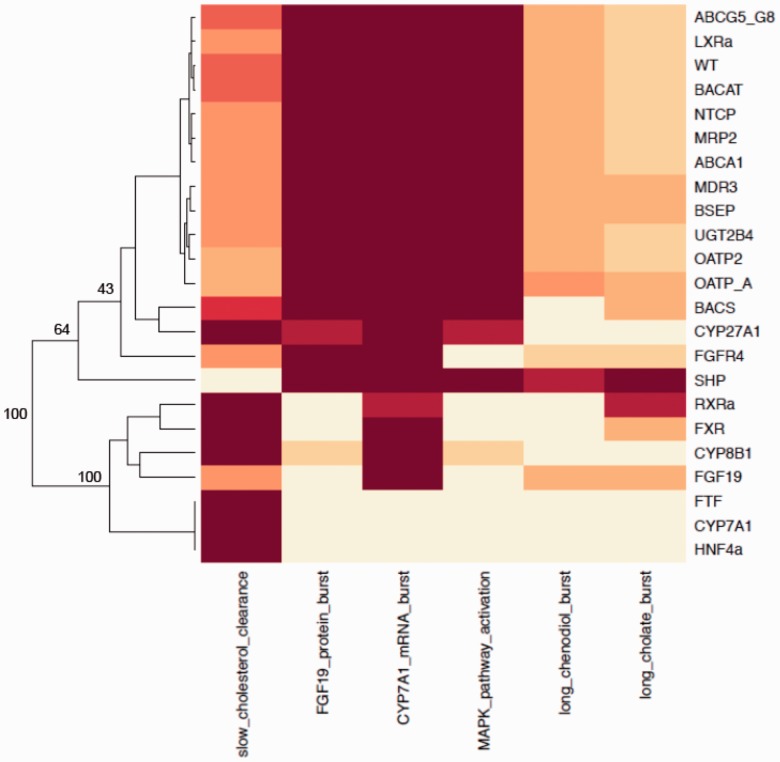
Clustering heatmap of computational knock-out results. Results of computational gene knock-down simulations of the model responding to cholesterol perturbation; 120 trajectories were run for each gene knock-out. Colour spectrum represents fraction of trajectories exhibiting behaviour of interest. Increasing colour intensity represents higher fraction of trajectories. The knock-down behaviour pairs where fractions are within 95% confidence intervals of WT are assigned the same colour as WT. Dendrogram shows result of complete-linkage clustering using Euclidean distance; bootstrap values (1000 iterations) are shown at major nodes. Monitored behaviours are defined in Supplementary Table S6.1. Significant deviation from wild type occurs in only 30/120 gene knock-out behaviour fields

Having demonstrated that the QSSPN framework can accurately reproduce a dynamic response to experimental perturbation in a human cell system, we went on to examine the genotype–phenotype relationship within the model system. We simulated single gene knock-outs of all genes represented in the DT set and evaluated their impact on the qualitative dynamic response to cholesterol perturbation. The initial conditions of simulations were set as before, with each of the genes sequentially knocked out by setting the number of tokens on the ‘inactive gene’ node to 0; for each *in silico* gene knock-out 120 trajectories were run. Subsequently, we calculated the fraction of trajectories showing six behaviours of interest designed to monitor BA homeostasis (Fig. 5).

This analysis shows that the system is robust and unsurprising, given its importance in BA homeostasis. The majority of single gene knock-outs did not result in significant departure from wild-type system behaviours, with only 23% of knock-out/behaviour comparisons showing behaviour probabilities outside of 95% binomial probability interval of a wild-type (WT). The analysis identifies a cluster of three genes, FTF, HNF4α and CYP7A1 that influence all behaviours to maximal extent. Knock-out of any of these genes results in all simulated trajectories exhibiting slow cholesterol clearance and no other behaviours. Our analysis also identifies SHP as a key regulatory molecule in BA homeostasis, as it is the only gene knock-out that significantly decreases the probability of slow cholesterol clearance. Such rapid cholesterol clearance is through the unregulated synthesis of BA, which could result in BA accumulation and toxicity. The mechanistic rationale underlying this observation is the fact that SHP transcription is regulated by FXR, which is activated by primary BAs. As SHP acts at the DR1 binding motif and inhibits production of BA synthesis enzymes, forming a negative feedback loop. Elimination of this feedback loop leads to a physiological imbalance in cholesterol clearance and disrupts the composition and concentration of the BA pool.

Simulations with our model also identify the coupling of the chenodiol and cholate branches of the BA synthesis pathway through the regulatory network. Knock-out of CYP8B1 affects both chenodiol and cholate homeostasis, despite only acting in the cholate branch of primary BA synthesis pathway. Simulations of CYP8B1 knock-out with the HepatoNet1 model using conventional CBM alone changes the maximal flux towards cholate from 0.898 to 0.1, without impacting on the maximal flux towards chenodiol. However, in our QSSPN model, CYP8B1 knock-out gene significantly decreases the number of trajectories in which both chenodiol and cholate rise to a maximal level. Examination of simulated trajectories and network connectivity shows that both branches of the BA biosynthesis pathway are coupled through the transcriptional regulatory network of nuclear receptors. Both primary BAs are FXR agonists and FXR activates the bileacyl-CoA synthetase (BACS) enzyme through an IR1 binding site in its promoter region. BACS impacts both chenodiol and cholate synthesis. Therefore, when CYP8B1 is inactivated, the maximal flux towards cholate decreases, decreasing the probability of FXR activation, BACS up-regulation and so affects the synthesis of both primary BAs. This result is a novel mechanistic insight into the clinically important process of BA synthesis, which could not have been obtained by FBA simulation of metabolic reaction network alone.

### 3.4 Software and model distribution

The QSSPN is implemented in C++ as a command line tool, extending our SurreyFBA software (Gevorgyan *et al.*, 2011). The CBM calculations are performed by SurreyFBA functions driven by the new code implementing PN data structure and QSSPN algorithm. Supplementary material provides the C++ source code and Mac OSX and Linux binaries of command line tool under GNU general public license (GPL). The models are built with Snoopy, a general graphic PN editor available as free software (Rohr *et al.*, 2010). The PN editor is used to create network connectivity of the DT part of the model, and QSSPN-specific parameters are contained within comments. One of the PN transitions represents the QSSF part of the model, which is fully described in an external file in SurreyFBA format. The SurreyFBA editor JyMet can be used to convert available GSMN reconstructions from SBML to SurreyFBA ,and thus QSSPN can benefit from the legacy of the ‘off the shelf’ genome-scale metabolic models. The extensible mark-up language file produced by Snoopy is converted to the input file of QSSPN command line tool by a Python script, which is also provided in supplementary material. The supplementary material provides hepatocyte model shown earlier in the text. The files in Snoopy and QSSPN formats can be used to reproduce simulations presented in this work and modify models. Network structure is also provided in PDF files, where vector graphics enable detailed examination of the connectivity. Finally, we have used Snoopy to generate SBML representation of the DT part of the hepatocyte model. The QSSF part of the model has been originally represented in SBML. The QSSPN rates and lookup tables are provided in the annotation sections of the SBML file. We believe that it is, in principle, possible to fully represent these QSSPN-specific features with SBML, but we consider this to be a subject of future development.

## 4 DISCUSSION

We present qualitative dynamic simulations of BA homeostasis in human hepatocytes incorporating gene regulation, signalling and whole-cell metabolism. This is the first dynamic simulation of molecular interaction networks describing gene regulation, signalling and whole-cell metabolism in human cells. We reproduce experimentally determined relative molecular activity timecourses and use the model to mechanistically investigate genotype–phenotype relationship in a clinically relevant system, which yields novel mechanistic insight. The presented mechanistic computer simulations are performed with QSSPN, a novel algorithm formulated in this work. QSSPN performs quasi-steady state simulation of genome-scale molecular interaction networks involving all classes of molecular interactions. Metabolic reactions are assumed to be much faster than other cellular processes and whole-cell, steady state, metabolic flux distribution is explored using well-established CBMs. Dynamic cellular processes are represented using extended PN formalism, and we introduce two new classes of PN node that integrate the steady state metabolic flux network and dynamic PN components of the model. Constraint nodes set the bounds of specified fluxes in the metabolic network according to a lookup table mapping its discrete token state to flux bounds. Objective nodes use predefined lookup tables to establish token state based on the evaluation of objective function in a whole-cell metabolic model. QSSPN is a stochastic simulation algorithm that generates a sample of dynamic trajectories permissible given the properties of the system under study and modelled stimuli. Thus, each trajectory represents a feasible sequence of molecular transitions. Trajectory sampling is used to study the effects of system perturbation (e.g. growth condition, gene knock-down, agonist/antagonist addition). Samples are generated for WT and perturbed states and the fraction of trajectories exhibiting a behaviour of interest is calculated for each state. QSSPN can be used to test not only simple reachability of a particular node of the network or the producibility of a particular metabolite, but also occurrence of dynamic behaviours (e.g. bursts and oscillations). We use piecewise linear definition of the stochastic transition propensity, where contribution of the pre-place (substrate, catalyst, inhibitor) to the propensity is determined by a lookup table with thresholds (Equations 1 and 2). This allows formulation of qualitative rules and enables simulations where complex qualitative dynamic behaviours can be generated in computationally efficient models representing molecular activities by a few discrete states.

Taking into account the features discussed earlier in the text, we believe that QSSPN is substantially different to existing approaches and offers numerous advantages in the modelling of genome-scale molecular interaction networks in human cells. In Section 7 of the supplementary material, we present detailed comparison with existing approaches and the remaining part of this discussion summarizes its conclusions. First, the only simulation of human metabolism in a quasi-steady state framework published so far (Krauss *et al.*, 2012) did not include gene regulation or signalling. The CBM model used the dFBA approach (Varma and Palsson, 1994) to integrate ODE models of drug concentration in physiological compartments. Currently, qualitative rule-based models of regulatory processes can be integrated with an FBA of metabolism using rFBA (Covert *et al.*, 2001). However, rFBA uses Boolean rules expressing relationships between network nodes representing genes. Importantly, transcription and translation processes are not distinguished and known mechanistic details about the nature of experimentally monitored or perturbed molecules cannot be expressed, both of which are achieved with QSSPN. We have created coarse-grained versions of our BA homeostasis model using the rFBA framework (Supplementary Material, Section 7). We show that to faithfully model human cell systems the original rFBA needs to be modified to remove reliance on maximization of biomass and simulation of cell culture growth. Furthermore, we show that even the best adaption of rFBA to simulation of human cell is still not able to achieve the predictive power of QSSPN (Table 1.) This is due to the fact that the rFBA gene regulatory network does not distinguish transcription and translation processes. As a result it fails to reproduce the qualitative behaviour of transcript activity in a negatively auto-regulated gene. QSSPN makes the correct prediction because of its representation of gene expression, which does not require quantitative parameters, but does resolve the two fundamental stages of gene expression.

**Table 1.** Summary of predictive power of rFBA and QSSPN approaches in comparisons between simulation and experimental data

Modelling Approach	rFBA no delay	rFBA2 constant delay	rFBA3 variable delay	QSSPN
Matthews correlation coefficient	0.360	0.360	0.543	0.817

Detailed quasi-steady state models, resolving mechanistic details of gene expression process, can be constructed with iFBA (Covert *et al.*, 2008) and idFBA (Min Lee *et al.*, 2008) methods, where CBMs are coupled to ODE models through quasi-steady state assumption. Alternatively, detailed quantitative models have been coupled to CBMs through a ‘diverse mathematics’ approach (Karr, 2012), where a tailored model for a particularly well-studied small model microorganism has been created. However, these methods require quantitative data to parameterize ODEs. For our test case study and other clinically relevant human systems such data are rarely available. Thus, the QSSPN is significantly better than other quasi-steady state methods in addressing the trade-off between detailed representation of molecular mechanisms and the requirement for quantitative data. We believe that this makes it ideally suited to modelling human systems, where accurate quantitative knowledge is more difficult to obtain, but qualitative molecular activity timecourses are readily measurable either by well-established qRT-PCR and western-blot assays or high-throughput ‘omics’ technologies. In Section 8 of the Supplementary Material, we analyze the computational efficiency of QSSPN and demonstrate that it is scalable to mechanistic modelling of GSMN in human cells.

The quasi-steady state methods developed to date are synchronous and deterministic; they produce one trajectory where at every step all rules are synchronously evaluated. By comparison, QSSPN is the first method that enables asynchronous, stochastic and rule-based simulations of a quasi-steady state system involving whole-cell metabolism. In every iteration, only one stochastic transition is generated, resulting in multiple alternative sequences of molecular interactions. This is an important advantage as biological systems are fundamentally asynchronous, meaning that asynchronous algorithms should be used for qualitative rule-based simulations (Remy *et al.*, 2003). Moreover, as QSSPN allows incorporation of quantitative rates, it offers the exciting prospect of building semi-quantitative models where individual trajectories are interpreted as different cellular behaviours occurring in a population of genetically identical cells because of stochastic phenotypic switching (Balaban *et al.*, 2004).

PN trajectory sampling in wild-type and perturbed systems has previously been used in the signalling PN approach (Ruths *et al.*, 2008a,b). For the first time, QSSPN uses PN trajectory sampling in the context of quasi-steady state simulations involving GSMNs. As a result, it hugely extends the scale of models that can be qualitatively studied. Moreover, signalling PN is based on the comparison of average simulation timecourses; although this can be done within the QSSPN approach, we recommend the implementation of an alternative approach developed in the model-checking field of computer science. Here, the numbers of trajectories exhibiting the behaviour of interest in the reference and perturbed systems are statistically compared: binomial probability confidence interval statistics have already been used in statistical model-checking software such as Prism (Kwiatkowska *et al.*, 2011). Finally, we use a piecewise-linear formulation of propensity functions. Piecewise-linear functions were previously used to provide a discrete qualitative equivalent of ODE models (Baldazzi *et al.*, 2010). Here, we use them for the first time to define propensity functions in a stochastic simulation algorithm. The stochastic model-checking approach is capable of analyzing arbitrary large-scale models, whereas previous applications of piecewise-linear functions were limited by algorithms based on generation of full reachability graph, which is usually too large to be computed for genome-scale networks.

In summary, we believe that QSSPN is a unique method for reconstruction and simulation of genome-scale molecular interaction network dynamics. It provides qualitative insight into system dynamics through qualitative simulation and enables mechanistic simulation of genotype–phenotype relationships in human cells, where quantitative data are sparse and qualitative molecular biology data are readily available. It is therefore an ideal approach for the iterative cycle of reconstruction, simulation and measurement that will eventually lead to the prediction of genotype–phenotype relationships in human tissues, through the mechanistic simulation of whole-cell molecular biology.

*Funding*: Biotechnology and Biological Sciences Research Council grant BB/I008195/1. AMK acknowledges support from Era SysBioPlus TB-HOST-NET grant (BB/I00453X/1).

*Conflict of interest:* none declared.

## Supplementary Material

Supplementary Data
