# Supplementary material for: QSSPN: dynamic simulation of molecular interaction networks describing gene regulation, signalling and whole-cell metabolism in human cells
Source: Bioinformatics. 2013 Sep 23;29(24):3181–90. doi: 10.1093/bioinformatics/btt552 (PMC3842758; doi:10.1093/bioinformatics/btt552)
Supplement: Supplementary Data [file supp_29_24_3181__index.html]

QSSPN: Dynamic Simulation of Molecular Interaction Networks Describing Gene Regulation, Signalling and Whole-Cell Metabolism in Human Cells. — QSSPN: dynamic simulation of molecular interaction networks describing gene regulation, signalling and whole-cell metabolism in human cells — QSSPN: dynamic simulation of molecular interaction networks describing gene regulation, signalling and whole-cell metabolism in human cells — QSSPN: dynamic simulation of molecular interaction networks describing gene regulation, signalling and whole-cell metabolism in human cells — Supplementary Data 

# QSSPN: dynamic simulation of molecular interaction networks describing gene regulation, signalling and whole-cell metabolism in human cells

## Supplementary Data

files

**Files in this Data Supplement:**

- Supplementary Data - pdf file
