# Supplementary material for: QSSPN: dynamic simulation of molecular interaction networks describing gene regulation, signalling and whole-cell metabolism in human cells
Source: Bioinformatics. 2013 Sep 23;29(24):3181–90. doi: 10.1093/bioinformatics/btt552 (PMC3842758; doi:10.1093/bioinformatics/btt552)
Supplement: Supplementary Data [file supp_btt552_QSSPN_SupplementaryText.pdf]

Supplementary Material

QSSPN: Dynamic Simulation of Molecular Interaction Networks Describing Gene Regulation, Signalling and Whole-Cell Metabolism in Human Cells.

C.P.Fisher, N.J.Plant, J.B.Moore and A.M.Kierzek*

Faculty of Health and Medical Sciences, University of Surrey, Guildford, Surrey GU2 7XH, UK.

1 FORMULATION OF QSSPN ALGORITHM 2
1.1 Petri Nets (PN) 2
1.2 Flux Balance Analysis (FBA). 3
1.3 QSSPN algorithm. 3
2 QSSPN HEPATOCYTE MODEL 7
3 QUALITATIVE MODEL PARAMETERS 8
4 COMPARISON OF RESULTS WITH EXPERIMENTAL DATA. 9
4.1 Definition of qualitative behaviours. 9
4.2 Comparison of simulated and experimentally observed qualitative behaviours. 10
4.3 Error function and predictive power of the model 18
5 THE CHOICE OF QUALITATIVE PARAMETERS. 21
6 DYNAMIC BEHAVIOURS IN GENE KNOCKOUT SIMULATIONS 23
7 COMPARISON OF QSSPN WITH EXISTING APPROACHES. 24
7.1 Comparison of QSSPN with Regulatory FBA (rFBA). 25
7.2 Conclusions. 33
8 COMPUTATIONAL EFFICIENCY OF QSSPN 34
9 SOFTWARE AND MODEL DISTRIBUTION. 36
9.1 Model files. 36
9.2 QSSPN model file format. 37
9.3 QSSPN control file format 38
9.4 Running a simulation. 39
9.5 Simulation output file format. 39
10 SUPPLEMENTARY REFERENCES 40

© Oxford University Press 2005

1 FORMULATION OF QSSPN ALGORITHM

In quasi steady state Petri net (QSSPN) simulation of molecular interaction network dynamics the interactions are divided into two sets: quasi-steady state fluxes (QSSF), representing metabolic reactions, and dynamic transitions (DT), representing other classes of molecular interaction, such as signalling pathways and gene regulation. We assume that following any state change of a regulatory network, the metabolic network quickly reaches quasi steady state, which is justified by the timescale separation of gene regulation and metabolism. The DT's are then represented using Petri Net (PN) formalism, while QSSF are modelled using Flux Balance Analysis (FBA). In the following sections we will define QSSPN algorithm. For the sake of presenting a full, self contained description of QSSPN, we will first re-introduce definitions of PN and FBA that can also be found elsewhere (e.g: Steggles et al. 2007, Lewis et. Al. 2012) and subsequently define our novel QSSPN algorithm.

1.1 Petri Nets.

A **standard Petri Net** is a directed bipartite graph, where two types of nodes represent **places** and **transitions**. Directed **edges** connect places to transitions and transitions to places. Since PN is a bipartite graph, there are no edges linking places to places and transitions to transitions. A place linked to a transition with a directed edge originating at the place is described as a **pre-place** of that transition. A place connected to a transition by a directed edge originating at the transition is described as a **post-place** of that transition. Each place of the PN may have a non-negative integer number of **tokens** associated with it. The distribution of tokens over all places in the PN is called **marking** and represents the state of the system. Upon **firing**, transitions change the state of the system by moving tokens from pre-places, to post-places. The number of tokens consumed from the given pre-place is specified by the **weight** of an edge linking the pre-place with the transition. The number of tokens added to the post-place is specified by the weight of the edge linking the transition and post-place. The transition may fire only when it is **enabled** (i.e. when all pre-places contain sufficient number of tokens, specified by a **firing rule)**. The PN formalism is associated with graphical notation, where places and transitions are represented by circles and rectangles respectively, edges are drawn as arrows and tokens are represented as dots or numbers drawn within place symbols (See Main Manuscript, Figure 1). The terminology above is summarised by the following commonly used definition:

$A\ standard\ Petri\ Net\ is\ a\ quadruple\ (P, T, F\ , m_0), where:$
- $P\ and\ T\ are\ disjoint\ finite\ sets\ of\ places\ and\ transitions\ respectively.$
- $F: ((P\times T) \cup (T\times P) \to \mathbb{N}_0\ is\ the\ set\ of\ directed\ arcs\ weighted\ by\ non\ negative\ integer\ numbers.$
- $m_0: P \to \mathbb{N}_0\ defines\ the\ initial\ marking.$

In the PN field, numerous extensions of the standard framework described above have been defined to increase its expressiveness. In QSSPN we use **extended Petri nets**, which are particularly useful in describing catalytic and inhibitory interactions in molecular networks. An extended PN adds two types of edges to the standard Petri Net. A **read edge** connects pre-place to the transition. The transition is enabled only if the pre-place connected by read edge has sufficient number of tokens. However, upon firing, the transition does not consume tokens from the pre-place. In graphical representation read edge is indicated by an arrow headed with a full circle. An **inhibitory edge** connects a pre-place to a transition. The transition is enabled only if the pre-place connected by inhibitory edge **is not** sufficiently marked. In graphical notation an arrow headed with an empty circle represents an inhibitory edge.

The dynamics of the PN are analysed by iteratively firing transitions, which change the marking. A set of firing rules associated with transitions and an algorithm used to generate a dynamic trajectory of the system defines the **Petri Net semantic**. In the simplest case, a transition is enabled when all pre-places connected by standard and read edges are sufficiently marked (i.e. have number of tokens larger than the weight of an edge connecting pre-place to transition) and all pre-places connected by inhibitory edges are not sufficiently marked (i.e. have token numbers lower than the weight of an edge connecting pre-place to transition). The dynamic trajectory is then generated by iteration of the following steps: i) identification of all enabled transitions, ii) random selection of one of the enabled transitions, iii) changing PN marking by firing of selected transition. However, many different semantics are available in PN tools and separation of semantics from connectivity is one of the most powerful features of the PN formalism, supporting testing of different modelling assumptions for the same network connectivity. For example, in continuous PNs, firing rules are expressed in terms of Ordinary Differential Equations (ODEs) and standard algorithms for numerical integration of ODEs are used to generate dynamic trajectories. Another example are stochastic PNs where firing rules are defined by probabilistic propensity functions and the Gillespie algorithm (Gillespie 1977) is used to generate a sample of dynamic trajectories. Definition of semantics in continuous and stochastic PNs requires the introduction of continuous, stochastic and immediate transition types, which, when enabled, are fired by different procedures. In QSSPN we define novel PN semantics. We define a piecewise-linear transition propensity function, which is particularly well suited for qualitative modelling (Equations 1 and 2 in Main manuscript, section 1.3 below). We then formulate QSSPN algorithm, which integrates PN updates with FBA simulations of the QSSF part of the model.

QSSPN uses PN to represent the DT part of the molecular interaction network. The places represent molecules and transitions represent molecular interactions. Read and inhibitory edges are particularly useful to express template, catalytic and inhibitory interactions where transition depends on the DNA/mRNA template, enzyme or inhibitor, but should not change pre-place state upon firing. The tokens represent discrete molecular activity levels. PN semantics are defined by the transition propensity function and hybrid simulation algorithm (Supplementary Figure 1.1) is used to explore dynamic behaviour of the DTs, in the context of whole-cell metabolic flux distribution simulated by FBA.

1.2 Flux Balance Analysis.

Flux Balance Analysis, frequently referred to as constraint-based method (CBM), explores global flux distribution in a metabolic network at steady state. The system of m coupled chemical reactions, involving n metabolites is defined as a stoichiometric matrix **S** with dimensions of m x n. Each value in the matrix represents the stoichiometric coefficient of a particular metabolite in a particular metabolic reaction. The stoichiometric coefficient is negative for substrates and positive for products. The stoichiometric matrix is sparse, since for most metabolite/reaction pairs the value of the stoichiometric matrix is 0, indicating that metabolite does not participate in the reaction. Vector **x** of length m assigns concentration to every metabolite and vector **v** of length n assigns metabolic flux to every reaction in the network. Time evolution of molecular concentrations in the system is given by the equation: $d\mathbf{x}/dt = \mathbf{Sv}$, which at steady state becomes $\mathbf{Sv} = \mathbf{0}$. This equation constrains the possible values of fluxes in vector **v**, for each metabolite the sum of fluxes producing this metabolite, must equal the sum of fluxes consuming the metabolite. Additional constraints are introduced by assigning **bounds** to every reaction: $\mathbf{f}_l < \mathbf{v} < \mathbf{f}_u$, where $\mathbf{f}_l$ and $\mathbf{f}_u$ are vectors of length n containing lower and upper reaction bounds, respectively. The bounds are used to constrain measured fluxes to experimentally known values and specify reaction reversibility. Reversible reactions can assume both positive and negative flux values, irreversible reactions are constrained to either positive or negative fluxes. Furthermore, the bounds are also used to define boundary conditions (i.e. the values of exchange fluxes, which are connected to sources and sinks of metabolic flux). The sources and sinks are frequently referred to as **external metabolites**, which do not have to be balanced. The external metabolites specify nutrients available and secreted in the medium.

In a genome scale metabolic network the flux values for most of the reactions are unknown, so the linear system given by equation $\mathbf{Sv} = \mathbf{0}$ cannot be solved for a unique vector **v**. Instead, FBA explores the space of flux distribution vectors **v** that satisfy steady state condition (the null space of **S**) and the bounds to test feasibility of metabolic functions of interest under given extracellular nutrient conditions. The following linear programming optimisation is performed:

$$\max_{\boldsymbol{f}_l,\boldsymbol{f}_u,\boldsymbol{S}} (\boldsymbol{c} \cdot \boldsymbol{v}), \mathit{such\ that}$$
$$\boldsymbol{S} \cdot \boldsymbol{v} = \boldsymbol{0}$$
$$\boldsymbol{f}_l < \boldsymbol{v} < \boldsymbol{f}_u$$

The linear programming (usually Simplex algorithm) is used to maximise **objective function**, which is formulated as a linear combination of fluxes in **v**. Thus, **c** is a vector of objective function coefficients set by the user. The use of linear programming guarantees finding maximal, unique value of objective function, but there may be many solution vectors **v** corresponding to this value. In its original formulation FBA has been used to calculate maximal growth rates of microbial cultures under steady state conditions. However, in the context of a human metabolic model, such as HepatoNet1 (Gille *et al.*, 2010) it is used to test feasibility of metabolic functions. If the unique, maximal value of objective function is 0, then a particular objective function is not feasible given stoichiometric constraints of 0. In QSSPN simulations presented in this work we use FBA to test whether metabolites in the network can be produced. Following the previous work of Imielinski and colleagues (Imielinski *et al.*, 2005) and ours (Bonde *et al.*, 2011) on "producibility plots", we set objective function coefficient vector **c** to the row of stoichiometric matrix corresponding to a metabolite, which is tested, and remove the balance requirement for this metabolite. In this way we maximise sum of fluxes connected to this metabolite. If maximal value of objective function is greater than 0, we conclude that metabolite can be produced by the system. We can also use this calculation to conclude that the flux towards the metabolite is greater under certain conditions rather than others. In this way we can use FBA to test whether the change of metabolic network flux bounds leads to increased availability of the metabolite.

1.3 QSSPN algorithm.

Here we describe the QSSPN algorithm that integrates PN and FBA to perform qualitative dynamic simulation of molecular interaction networks involving gene regulation, signalling and whole cell metabolism. For the sake of the completeness of this description, we repeat Equations 1 and 2 of the main manuscript, which are key for defining PN semantics used in our qualitative simulations. Subsequently, we formulate a number of functions describing elementary operations within one algorithm iteration. These functions are then used on Supplementary Figure 1.1 that presents QSSPN as an algorithm flowchart.

1.3.1 Transition propensity function and transition types.

The propensity of PN transition t, denoted by P_t, is evaluated using the following formula (Equation 1 in Main Manuscript):

$$P_t = c_t \prod_{i=1}^{N} \mu_i(x_i)$$

where c_t is a rate constant, N is the number of pre-places of transition t, x_i is the number of tokens at pre-place i and μ_i is the pre-place activity in the transition depending on the pre-place state. The activity function is a look-up table of T thresholds t_i and activities a_i allowing general definition of the pre-place contribution to the transition propensity (Equation 2 in Main Manuscript):

$$\mu(x) = \left\{ \begin{array}{c} x \in [t_1, t_2), \quad \mu(x) = a_1 \\ x \in [t_i, t_{i+1}), \quad \mu(x) = a_i \\ \\ \ldots \\ x \in [t_{T-1}, t_T), \quad \mu(x) = a_T \end{array} \right\}$$

The interpretation of a transition propensity during simulations is dependent on the transition type, either **stochastic**, **continuous** or **immediate**. Stochastic transition propensity is interpreted as the probability density of the transition firing in the next, infinitesimally small, time step. Continuous transitions are updated after each iteration of the simulation and the number of tokens moved is proportional to the propensity (i.e. the propensity is used as reaction rate). Finally, immediate transitions fire whenever their propensity function is different than 0. Regardless of transition type, the transition with propensity of 0 is not enabled and it does not move any tokens.

1.3.2 Setting of flux bounds (setQSSFbounds())

This function sets the bounds of fluxes in the quasi-steady state flux (QSSF) part of the model according to the state of the **constraint node**. Each constraint node is associated with a list of fluxes in the QSSF network and a lookup table setting bounds of all fluxes from this list according to the node's state. The lookup table has the following form:

$$(lb(x), ub(x)) = \left\{ \begin{array}{c} x \in [t_1, t_2), \quad lb(x) = f_{l,1},\, ub(x) = f_{u,1} \\ x \in [t_i, t_{i+1}), \quad lb(x) = f_{l,i},\, ub(x) = f_{u,i} \\ \\ \ldots \\ x \in [t_{T-1}, t_T), \quad lb(x) = f_{l,T},\, ub(x) = f_{u,T} \end{array} \right\}$$

where x is the state of the node (number of tokens), lb(x), ub(x) are lower and upper flux bounds respectively, $t_1,..,t_T$ are number of tokens thresholds and $f_{l,i}$, $f_{u,i}$ are lower and upper flux values assigned according to the state of the node. If application of the lookup table results in a change to the lower or upper bound for any of the fluxes, the global Boolean variable 'UpdateRequired' is set to TRUE to indicate that flux balance analysis (FBA) objective functions need to be evaluated. The UpdateRequired variable is re-set to FALSE before the loop over constraint nodes commences.

1.3.3 Evaluation of an objective function (evaluateObjective())

This function uses Flux Balance Analysis to evaluate the objective function specified by a particular **objective node**. The objective function is specified as the name of the flux or the name of the metabolite in the QSSF network. If the objective is specified as the flux the linear programming (LP) maximises value of this flux. If the objective is specified as a metabolite, the sum of fluxes producing this metabolite (metabolite producibility (Imielinski *et al*., 2005; Bonde *et al*., 2011)) is maximised. If 'UpdateRequired' variable is set to TRUE the objective is evaluated by LP maximisation of entire QSSF model. Otherwise, the value determined for this objective in the previous iteration is used. Evaluation of an objective function by LP maximisation of the entire QSSF model is the most computationally demanding step of the QSSPN algorithm. The use of 'UpdateRequired' variable ensures that this step is not executed unnecessarily, when QSSF bounds have not changed, and the previously determined value of the objective function would be obtained.

1.3.4 Setting of objective node state (updateObjectiveNode())

This function sets the state of a particular objective node according to the objective function value, thus feeding back information about steady state metabolic capabilities to the dynamic part of the model. The following lookup table is used to set the state of the objective node:

$$x(o) = \left\{ \begin{array}{c} o \in [t_1, t_2), \quad x(o) = s_1 \\ o \in [t_i, t_{i+1}), \quad x(o) = s_i \\ \\ \ldots \\ o \in [t_{T-1}, t_T), \quad x(o) = s_T \end{array} \right\}$$

where x(o) is the state of the objective node, $t_1,..,t_T$ are objective function thresholds (real numbers) and $s_1,..,s_T$ are integer number of tokens assigned to the node, depending on the objective function value *o* calculated by evaluateObjective().

1.3.5 Firing of continuous and immediate transitions (fireDeterministicTransitions(Δt))

The propensity function of each continuous and immediate transition is evaluated according to Equation 1 (main text). Subsequently, each immediate transition for which propensity function is greater than 0 is fired once. For each continuous transition the number of firings k is evaluated as a product of propensity function and timestep **Δ**t rounded to the nearest integer. The continuous transition is then fired k times. All node state updates within this function are executed synchronously. For each pre-place and post-place associated with continuous or immediate transition the change of state resulting from transition firings is calculated first. Subsequently, the state of the system is updated by adding node state changes to the node states.

1.3.6 Firing of delayed transitions (fireDelayedTransition(t, t))

This function returns a Boolean value; the function checks if there are any delayed transitions to be fired in the time interval (t, t+t). If there are no delayed transitions scheduled to fire the function returns FALSE. Otherwise, it fires one delayed transition and sets simulation time t to the time t_s at which this transition has been scheduled. The **Δ**t parameter is then set to $t_s – t$. If there are multiple delayed transitions set to fire in the time interval (t, t+t) the transition that is scheduled at the earliest time is fired. After firing the transition the function returns TRUE.

1.3.7 Scheduling of delayed transitions (scheduleDelayedTransition(m))

If the stochastic transition m selected to be fired has delay time t_d greater than 0 this function adds the transition to the list of delayed transitions to be fired. The transition is scheduled to fire at time $t_s = t + t_d$.

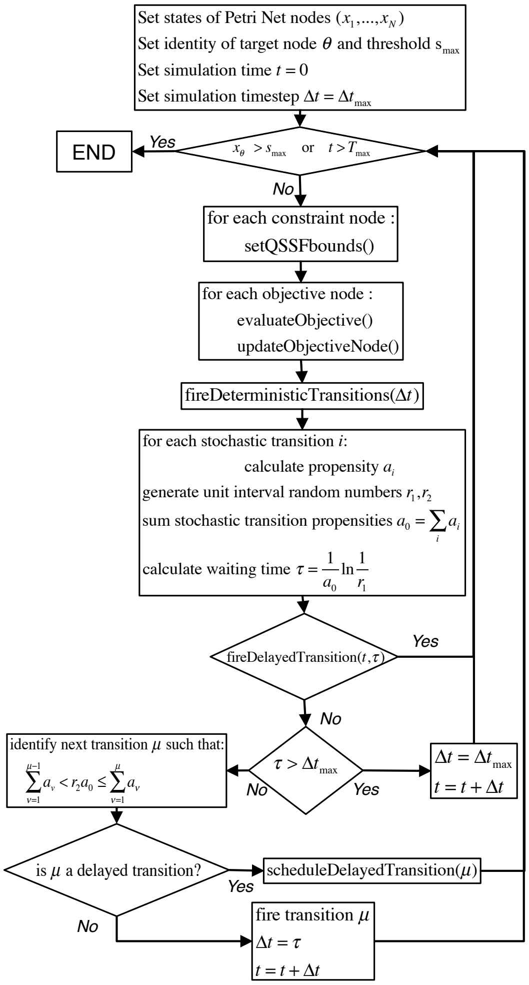

Supplementary Figure 1.1. QSSPN algorithm flowchart.

1.3.8 Hybrid simulation algorithm.

The flowchart of QSSPN simulation algorithm is shown on Supplementary Figure 1.1. The QSSPN simulation is performed by a hybrid simulation algorithm based on the maximal timestep method (Puchalka & Kierzek 2004), previously used to build quantitative, stochastic, kinetic models of systems involving reactions with rates differing by many orders of magnitude. The method uses the current timestep, Δt, which is smaller than the maximum timestep, Δt_{max}. First, QSSF bounds are updated according to the state of constraint nodes. The state of each objective node is then determined through FBA maximisation of the objective function associated with that node. Subsequently, continuous and immediate transitions are updated; propensities of these transitions are calculated according to the current state of the system and each immediate transition for which propensity is greater than 0 is fired once. Continuous transitions are updated by executing the Euler algorithm: the number of transition firings is determined as the nearest integer to the product of the propensity function and Δt and the transition is fired this number of times. Finally, one of the stochastic transitions is fired according to the Gillespie algorithm (Gillespie 1977); the propensity of all stochastic transitions is calculated, the transition to be fired next is randomly selected and the waiting time randomly generated. The probability of a transition being selected is proportional to its propensity function. Waiting times are exponentially distributed with the rate parameter equal to the sum of transition propensities. If the waiting time is smaller than the Δt_{max} then the selected transition is fired once, simulation time advanced by the waiting time and Δt set to the waiting time. Otherwise, no stochastic transition is fired and Δt is set to Δt_{max}. Simulation time is then advanced by adding Δt to the current time. To further enhance the power of our method in expressing knowledge about molecular interactions, we allow stochastic transitions to be delayed (i.e. scheduled to fire at a certain time after the firing time determined by the Gillespie algorithm). Iteration of the above steps results in the generation of a single dynamic trajectory of the system, which, following PN nomenclature, will be referred to as a token game trajectory.

2 QSSPN HEPATOCYTE MODEL

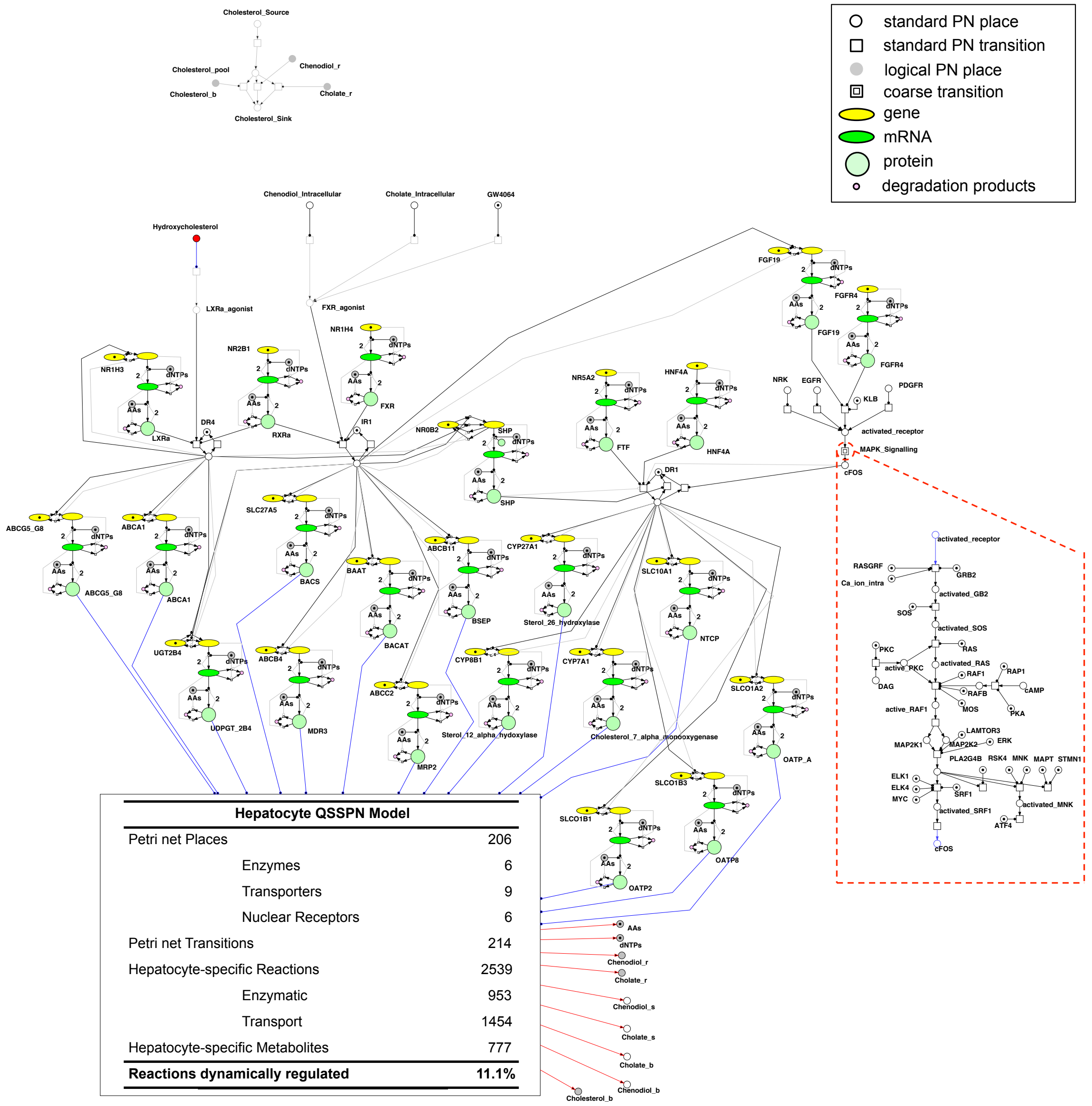

Hepatocyte QSSPN Model	
Petri net Places	206
Enzymes	6
Transporters	9
Nuclear Receptors	6
Petri net Transitions	214
Hepatocyte-specific Reactions	2539
Enzymatic	953
Transport	1454
Hepatocyte-specific Metabolites	777
**Reactions dynamically regulated**	**11.1%**

SUPPLEMENTARY FIGURE 2.1. The QSSPN model of global hepatocyte metabolism regulating bile acid homeostasis in human liver. The QSSPN network was built using the Snoopy Petri net editor. The HepatoNet1 genome scale metabolic network model is encapsulated within a large transition symbol. Constraint nodes are connected to the metabolic network by read edges coloured in blue. Metabolic network is connected to objective nodes by edges coloured in red. The mitogen activated protein kinase (MAPK) signalling cascade is encapsulated within a coarse transition symbol, shown on the network map in both a compressed and expanded format.

3 QUALITATIVE MODEL PARAMETERS

SUPPLEMENTARY TABLE 3.1. Qualitative parameters used in human hepatocyte model

Name	Value	Description
**General QSSPN parameters applied to all nodes, transitions and edges unless stated otherwise below**		
c_1	1.0	*Default rate constant.* Parameter c_t in Equation 1, main text.
N	2	*Default maximal number of tokens*. By default three state model was used where the PN node can have 0, 1 or 2 tokens.
$\mu_1(x)$	$\begin{cases} x \in [0,1) & \mu_1(x) = 0.0 \\ x \in [1,2] & \mu_1(x) = 1.0 \end{cases}$	*Default activity table of edge and read edge.* See Equation 2 in main text for definition of activity table.
$\mu_2(x)$	$\begin{cases} x \in [0,1) & \mu_2(x) = 1.0 \\ x \in [1,2] & \mu_2(x) = 0.0 \end{cases}$	*Default activity table of inhibitory edge.* If the pre-place has more than 1 token, transition propensity is multiplied by 0.
**Parameters of general gene expression model (see main text) used for all genes unless stated otherwise below**		
c_2	100.0	*Promoter activation rate.*
c_3	immediate	*Promoter inactivation rate.*
$\mu_3(x)$	$\begin{cases} x \in [0,2) & \mu_3(x) = 1.0 \\ x = 2 & \mu_3(x) = 0.0 \end{cases}$	*Synthesis/degradation rule.* The activity table of inhibitory edge preventing unnecessary firing of transcription/translation reactions when mRNA/protein reach state of 2 tokens.
c_4	immediate	*Binding site activation.*
c_5	10.0	*Binding site inactivation rate.*
c_6	immediate	*Transcription.*
c_7	immediate	*Translation.*
c_8	10.0	*Basal activation rate of positively autoregulated promoter.*
$\mu_4(x)$	$\begin{cases} x \in [0,1) & \mu_4(x) = 0.0 \\ x = 1 & \mu_4(x) = 10.0 \end{cases}$	*Activity of transcription factor in activation of its own promoter.*
c_9	10.0	*Inactivation rate of positively autoregulated promoter*
**Constraint and objective node parameters specific to HepatoNet1 metabolic network with PIPES boundary conditions**		
(lb_{irrev}, ub)	$\begin{cases} x \in [0,1) & lb_{irrev}(x) = 0.0 & ub(x) = 0.1 \\ x \in [1,2) & lb_{irrev}(x) = 0.0 & ub(x) = 0.5 \\ x = 2 & lb_{irrev}(x) = 0.0 & ub(x) = 1.0 \end{cases}$	*Bounds of irreversible reactions.*
(lb_{rev}, ub)	$\begin{cases} x \in [0,1) & lb_{rev}(x) = -0.1 & ub(x) = 0.1 \\ x \in [1,2) & lb_{rev}(x) = -0.5 & ub(x) = 0.5 \\ x = 2 & lb_{rev}(x) = -1.0 & ub(x) = 1.0 \end{cases}$	*Bounds of reversible reactions.*
N_1	10	*Maximal number of tokens for objective node.*
$x_1(o)$	$\begin{cases} o \in [0, 0.001) & x_1(o) = 0 \\ o \in [0.001, 0.09) & x_1(o) = 1 \\ o \in [0.09, 0.2) & x_1(o) = 2 \\ o \in [0.2, 0.3) & x_1(o) = 3 \\ o \in [0.3, 0.4) & x_1(o) = 4 \\ o \in [0.4, 0.5) & x_1(o) = 5 \\ o \in [0.5, 0.6) & x_1(o) = 6 \\ o \in [0.6, 0.7) & x_1(o) = 7 \\ o \in [0.7, 0.8) & x_1(o) = 8 \\ o \in [0.8, 0.9) & x_1(o) = 9 \\ o > 0.9 & x_1(o) = 10 \end{cases}$	*Mapping of objective function values to objective node state.*
**Parameters specific to Hepatocyte model**		
c_{10}	0.1	*SHP protein degradation rate.*
c_{11}	0.1	*SHP promoter basal activation rate.*
$\mu_5(x)$	$\begin{cases} x \in [0,1) & \mu_5(x) = 1.0 \\ x = 1 & \mu_5(x) = 1000.0 \end{cases}$	*Activity of bound DR4 and IR1 in activation of SHP promoter.* If the binding sites are not bound (x=0) the activity is set to 1 and propensity of promoter activation equals 0.1. If binding site is bound the promoter activation rate equals 100, a default for all promoters.
$x_2(o)$	$\begin{cases} o \in [0, 0.001) & x_2(o) = 0 \\ o > 0.001 & x_2(o) = 1 \end{cases}$	*Mapping of dNTP and AAs objective function to the state of objective node.* We required that the flux towards mRNA and protein building blocks is larger than 0. We did not monitor value of this flux with any more detail.
$\mu_6(x)$	$\begin{cases} x \in [0,3) & \mu_6(x) = 0.0 \\ x \geq 3 & \mu_6(x) = 1.0 \end{cases}$	*Threshold for presence of LXR and FXR agonist.*
d_1	5.0	*Delay of FGF19 binding to FGF1 receptor.*

4 COMPARISON OF RESULTS WITH EXPERIMENTAL DATA.

To evaluate the predictive power of our model, we have compared simulation results with published experimental data (Song *et al.*, 2008). This work is representative of typical molecular biology data, where relative changes in transcript expression over time or in response to experimental perturbation are determined. In section 4.1, we define qualitative behaviours used to describe both experimental and simulation results. In section 4.2, we summarise the experimental findings and directly compare these data with QSSPN simulations performed using our qualitatively prameterised model (supplementary figure 2.1, supplementary table 3.1). Subsequently, in sections 4.3 and 4.4 we formulate an error function and use it to evaluate predictive power of our model. In section 4.5 we conclude that QSSPN simulation is capable of predicting qualitative system behaviours for important molecular systems of interest, where information on quantitative rate constants and molecular amounts is limited.

4.1 Definition of qualitative behaviours.

In order to compare experimental data, digitised from the figures of Song *et al* 2008, with simulation results we apply the model checking approach of computer science, defining qualitative dynamic behaviours which can be formally attributed to both experimental and simulated time-courses (Kwiatkowska *et al.*, 2011). We start simulations at initial conditions reflecting the design of laboratory experiments and subsequently compare the qualitative behaviours exhibited by simulation results and experimental data. We believe this approach to qualitative modelling to be better than the alternative of matching simulation and experimental time-points to compare measured and simulated values at each time-point. As many of the quantitative rate constants are not available for our modelled system, simulation time is qualitative and expressed in arbitrary time units. Thus, simulation time serves to determine which events happened before, after or simultaneously with another transition, but the intervals between events do not correspond to real time units. As such, matching time-points would be a difficult and arbitrary exercise, especially considering that experimental time-points are relatively sparse and the precise time of molecular activity rises and falls is not accurately known. For example, in the experimentally determined SHP transcript time-course (Supplementary Figure 4.3), relative transcript levels can potentially return to basal level at any time between 6 and 24 hours. Instead, we define the qualitative behaviour exhibited over an entire time-course. For example, activation is defined as the rise of a molecular species to a certain level, which is then maintained until the end of the time-course. The exact time of this rise does not matter; we only require that it is lower before the rise and that it remains at a higher level at all time-points after the rise, thus distinguishing it from a single burst or oscillation.
Apart from the analysis of timecourse data (Supplementary Figures 4.1-4.6), this model checking approach can also be used to analyse data on the relative activity of molecular species at selected, biologically meaningful, timepoints. Supplementary Figures 4.7-4.10, show this common type of experimental data. The timecourse data was first used by Song *et al*. to determine at which timepoint biological effects of interest occur and subsequently, relative transcript levels were measured at this timepoint for a range of perturbations. We demonstrate here, and in section 7, that modelling this type of data by the number of trajectories exhibiting a behaviour of interest, as determined by Monte Carlo sampling, in both a perturbed and control system is one of the advantages of QSSPN over alternative approaches.

Below, we define eight qualitative behaviours to compare experimental and simulation results; these definitions are applicable to both experimental and simulated data. The "relative amount" refers to token number in simulations and signal ratio in experimental data. The "activity" refers to the number of trajectories exhibiting the behaviour of interest in simulations and the signal at single timepoint in experimental data. A "significant difference" is declared where Monte Carlo sampling or experimental data error bars do not overlap. The 95% binomial probability confidence interval was used for calculation of simulation error bars and experimental error bars reported in original publication of *Song et al* were used for experimental data.

Activation: *Let L_0 be the relative amount of the molecular species at first timepoint of the timecourse. The timecourse exhibits activation if the relative amount rises above L_0 and remains larger than L_0 until the end of the timecourse.*

Inhibition: *Let L_0 be the relative amount of the molecular species at first timepoint of the timecourse. The timecourse exhibits inhibition if the relative amount falls below L_0 and remains smaller than L_0 until the end of the timecourse.*

Burst: *Let L_0 be the relative amount of the molecular species at first timepoint of the timecourse. The timecourse exhibits burst if the relative amount rises above L_0, returns to L_0 and remains equal to L_0 until the end of the timecourse.*

Constant: *Let L_0 be the relative amount of the molecular species at first timepoint of the timecourse. The timecourse exhibits constant behaviour if the relative amount equals L_0 at every timepoint of the timecourse.*

Oscillation: *Let L_0 be the relative amount of the molecular species at first timepoint of the timecourse. The timecourse exhibits oscillation if the relative amount rises above L_0, returns to L_0 and then rises above L_0 again before end of the timecourse.*

Increase: *The activity of a molecular specise in the treated system is significantly higher than in the control system.*

Decrease: *The activity of a molecular species in the treated system is significantly lower than in the control system.*

Equal: *The activity of a molecular species in the treated system is not significantly different from its activity in the control.*

4.2 Comparison of simulated and experimentally observed qualitative behaviours.

In this section we present twelve direct comparisons of the qualitative behaviours determined by the experiments of Song *et al*. and our simulations. For each of these comparisons we reproduce the published experimental data, and our simulation results. Subsequently, we present two simulations designed to test whether the system is capable of maintaining primary bile acid homeostasis – a major, well documented, physiological function of hepatocytes (Chiang, 2009). These comparisons, of simulation results with published qualitative behaviours, will be used to evaluate the predictive power of the model, compare QSSPN with other approaches and assess the robustness of simulation results to the perturbation of numerical value of key qualitative parameters.

Simulations were started from an initial state where 1 token was present at each place representing an inactive gene and 0 tokens were assigned to all other places; this initial state represents the genotype of the system. Each trajectory of the QSSPN simulation was run as follows. The simulation was run for 1 arbitrary time unit to allow all the genes to express their products under conditions of no perturbation, the GW4064 node was then assigned one token and simulation continued for another 10 time units. This reflects the conditions in a biological experiment (perturbation is applied to the system with the given genotype expressed under condition of no perturbation). For example, Supplementary Figure 4.2 shows CYP7A1 transcript constitutively expressed under condition of no perturbation and then inhibited following treatment with GW4064. The opposite situation is shown in Supplementary Figure 4.1; the FGF19 transcript is not expressed until activated by treatment. Thus, our protocol, initiating simulations from a set genotype and allowing the system to achieve normal basal expression prior to perturbation allows robust modelling of a typical *in vitro* cell culture experiment.

One hundred and twenty individual trajectories using the simulation protocol described above were run. Each trajectory was examined to check whether it exhibited activation, inhibition, burst, constant or oscillation behaviour according to the definitions from Chapter 4.1. The number of trajectories exhibiting each of these behaviours were calculated. In accordance with the model checking literature (Kwiatkowska *et al.*, 2011), we have calculated the fraction of trajectories exhibiting defined behaviours and 95% binomial probability confidence intervals to assess the accuracy of our estimates for the Monte Carlo sample size of 120 (binconf function of R Hmisc package). Final conclusion of the simulation was based on the value of lower confidence interval. The simulation was stated to exhibit a particular behaviour for a given node (molecule) of interest (e.g. activation of FGF19_mRNA) if the lower 95% confidence interval calculated for this node was larger than 0.5 (i.e. 95% confidence that more trajectories in the sample exhibit a given behaviour than any other).

4.2.1 Activation of FGF19 following GW4064 treatment.

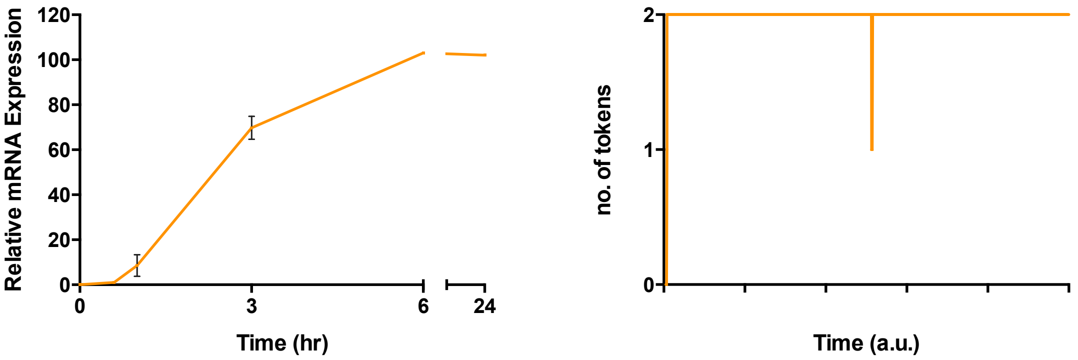

Supplementary Figure 4.1. Activation of FGF19 transcript following GW4064 treatment. Experimental data from Figure 1A of Song *et al*. 2008 (left) and example token game trajectory (right). Both exhibit the same qualitative, activation behaviour.

Supplementary Table 4.1. Results obtained for FGF19 transcript (FGF19_mRNA node).

**Most Frequently Occurring Behaviour**	**Activation**
Number of trajectories	117
Fraction of trajectories	0.975
Lower 95% CI	0.92907
Upper 95% CI	0.9914618

We conclude that in this simulation FGF19 transcript exhibits activation behaviour, in agreement with experimental timecourse data (Supplementary Figure 4.1).

4.2.2. Inhibition of CYP7A1 transcript following GW4064 treatment.

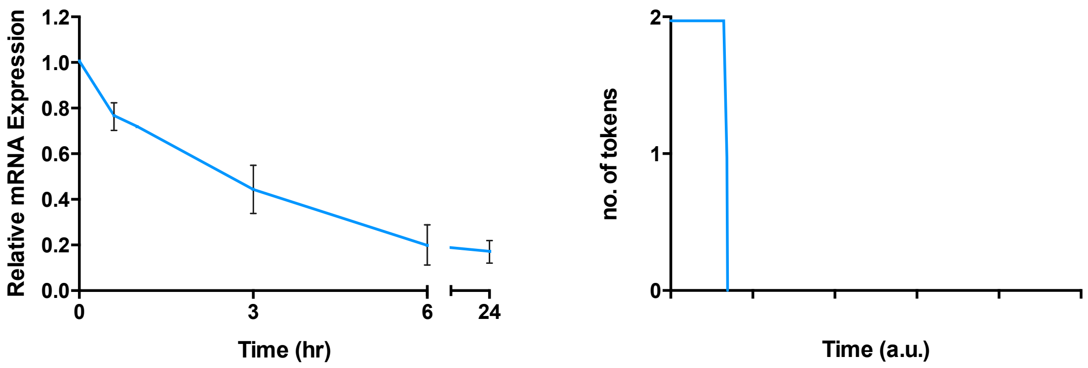

Supplementary Figure 4.2. Inhibition of CYP7A1 transcript following GW4064 treatment. Experimental data from Figure 1A of Song *et al*. 2008 (left) and example token game trajectory (right). Both exhibit the same qualitative, inhibition behaviour.

Supplementary Table 4.2. Results obtained for the CYP7A1 transcript (CYP7A1_mRNA node).

Most Frequently Occurring Behaviour	Inhibition
Number of trajectories	100
Fraction of trajectories	0.8333333
Lower 95% CI	0.7565472
Upper 95% CI	0.88944

We conclude that in this simulation, CYP7A1 transcript exhibits inhibition behaviour, in agreement with experimental timecourse data (Supplementary Figure 4.2).

4.2.3 Burst of SHP transcript following GW4064 treatment.

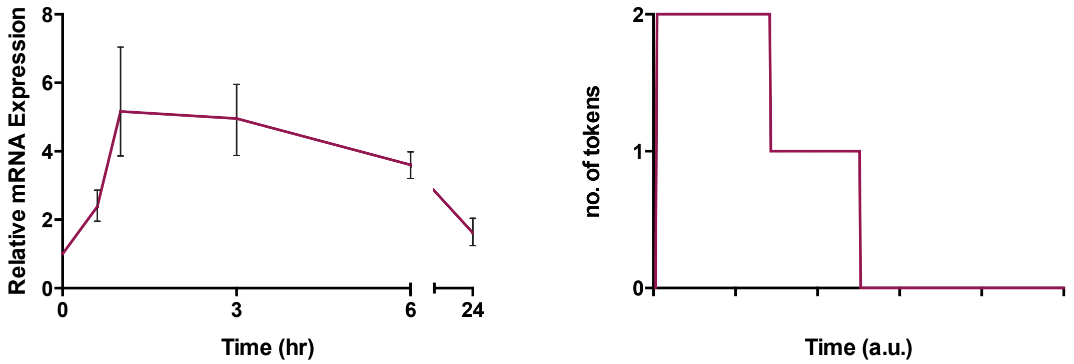

Supplementary Figure 4.3. Burst of SHP transcript following GW4064 treatment. Experimental data from Figure 1A of Song *et al*. 2008 (left) and example token game trajectory (right). Both exhibit the same qualitative, burst behaviour.

Supplementary Table 4.3. Results obtained for the SHP transcript (NR0B2_mRNA node).

Most Frequently Occurring Behaviour	Burst
Number of trajectories	79
Fraction of trajectories	0.6583333
Lower 95% CI	0.5697483
Upper 95% CI	0.7370956

We conclude that in this simulation, SHP transcript exhibits bursts behaviour, in agreement with experimental timecourse data (Supplementary Figure 4.3).

4.2.4 Constant level of HNF4a following GW4064 treatment.

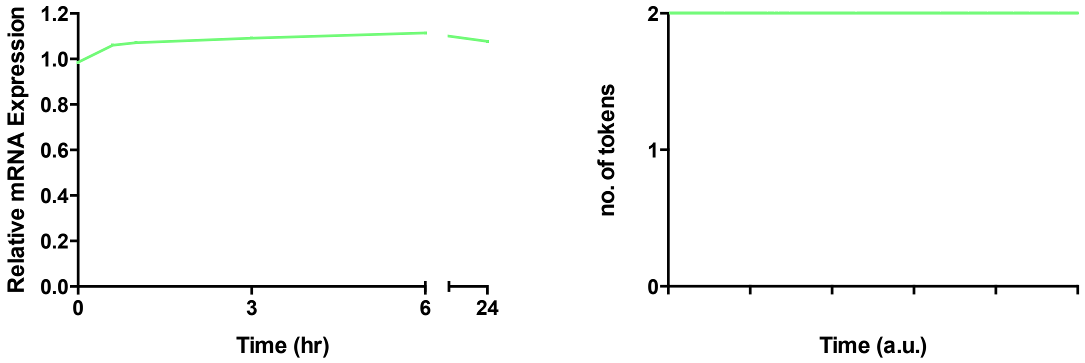

Supplementary Figure 4.4. Constant level of HNF4α transcript following GW4064 treatment. Experimental data from Figure 1A of Song *et al*. 2008 (left) and example token game trajectory (right). Both exhibit the same qualitative, constant behaviour.

Supplementary Table 4.4 Results obtained for the HNF4 α transcript (HNF4A_mRNA node).

Most Frequently Occurring Behaviour	Constant
Number of trajectories	120
Fraction of trajectories	1.0
Lower 95% CI	0.9689808
Upper 95% CI	1.0

We conclude that in this simulation, HNF4α transcript exhibits constant behaviour, in agreement with experimental timecourse data (Supplementary Figure 4.4).

4.2.5 Inhibition of CYP7A1 transcript by FGF19.

Simulations were run using the same protocol as previously described, but with a different perturbation. After simulation time of one arbitrary time unit, the FGF19 protein (FGF19 node) was permanently set to a state of 2 tokens.

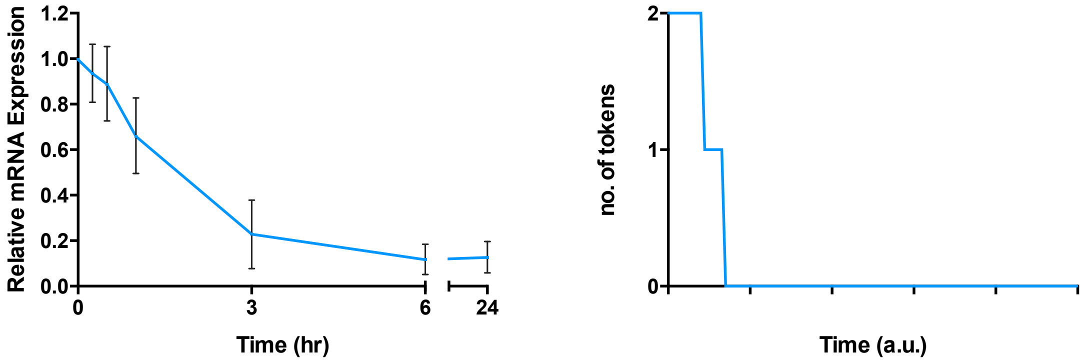

Supplementary Figure 4.5. Inhibition of CYP7A1 transcript by FGF19. Experimental data from Figure 1A of Song *et al*. 2008 (left) and example token game trajectory (right). Both exhibit the same qualitative, inhibition behaviour.

Supplementary Table 4.5. Results obtained for the CYP7A1 transcript (CYP7A1_mRNA node).

Most Frequently Occurring Behaviour	Inhibition
Number of trajectories	112
Fraction of trajectories	0.9333333
Lower 95% CI	0.8739487
Upper 95% CI	0.9658347

We conclude that in this simulation, CYP7A1 transcript exhibits inhibition behaviour, in agreement with experimental timecourse data (Supplementary Figure 4.5).

4.2.6 Burst of SHP transcript following FGF19 treatment.

Simulations were run using the same protocol as previously described, but with a different perturbation. After simulation time of one arbitrary time unit, the FGF19 protein (FGF19 node) was permanently set to a state of 2 tokens.

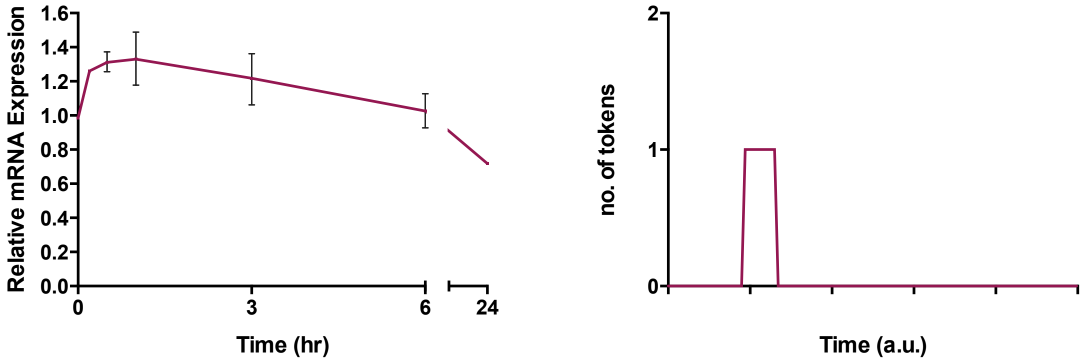

Supplementary Figure 4.6. Burst of SHP transcript by FGF19. Experimental data from Figure 3C of Song *et al*. 2008 (left) and example token game trajectory (right). Both exhibit the same qualitative, burst behaviour.

Supplementary Table 4.6. Results were obtained for the SHP transcript (NR0B2_mRNA node).

**Most Frequently Occurring Behaviour**	**Burst**
Number of trajectories	82
Fraction of trajectories	0.6833333
Lower 95% CI	0.5955213
Upper 95% CI	0.7597717

We conclude that in this simulation, SHP transcript exhibits burst behaviour, in agreement with experimental timecourse data (Supplementary Figure 4.6). While the SHP burst is smaller in magnitude than the one shown in Supplementary Figure 4.3, the first and final two timepoints of the experimental data are below the error bars of the highest timepoint. Thus, the experimental timecourse fulfils definition of burst behaviour.

4.2.7. FGF19 treatment decreases relative CYP7A1 expression.

Supplementary Figure 4.7 shows experimental data on the change in CYP7A1 transcript levels after perturbation, relative to appropriate controls. We have developed the following simulation protocol to replicate this common experimental design. First, simulations were run for untreated control. The 120 trajectories were started from an initial state representing the genotype, where 1 token was present at each place representing inactive gene and 0 tokens were assigned to all other places. The number of trajectories exhibiting CYP7A1 transcript activation was calculated. The simulation time was set such that the fraction of trajectories exhibiting the behaviour of interest in control simulations was significantly greater than 0 and less than 1. This enabled the subsequent evaluation of whether the fraction of trajectories exhibiting CYP7A1 activation in the treated system was higher or lower than in controls. Using this approach, setting a simulation time where the fraction of trajectories is 0.5 in the control system would be ideal, but is difficult to achieve exactly. A simulation time of 5.0 arbitrary units was chosen for which the fraction of trajectories exhibiting CYP7A1 transcript activation was 0.6. Subsequently, simulations were run with different perturbations, using the same simulation time, and the fraction of trajectories exhibiting CYP7A1 activation compared with the control. The fractions, for which the 95% binomial probability confidence intervals did not overlap, were considered to be significantly different. The behaviour of the treated system, relative to the control, was then classified as ‘increase’, ‘decrease’ or ‘equal’ as defined in section 4.1.

We would like to note that the computer simulation protocol described above reflects experimental procedures. To obtain the experimental data shown in Supplementary Figure 4.7, Song *et al* would have first examined timecourse data of CYP7A1 activation and chosen 6 hours as a suitable treatment time to test upregulation or downregulation of CYP7A1 transcript. In our computer simulation protocol, we examine trajectories in a control experiment to determine optimal simulation time to examine the effect of a perturbation. Thus, we identify an appropriate simulation time to test the potential increase or decrease in the number of behaviour occurrences with respect to the control. In section 7 we show that using Monte Carlo sampling of trajectories, in this manner, provides a unique advantage over other qualitative simulation methods.

In this simulation, FGF19 treatment was implemented in the same way as in section 4.2.5.

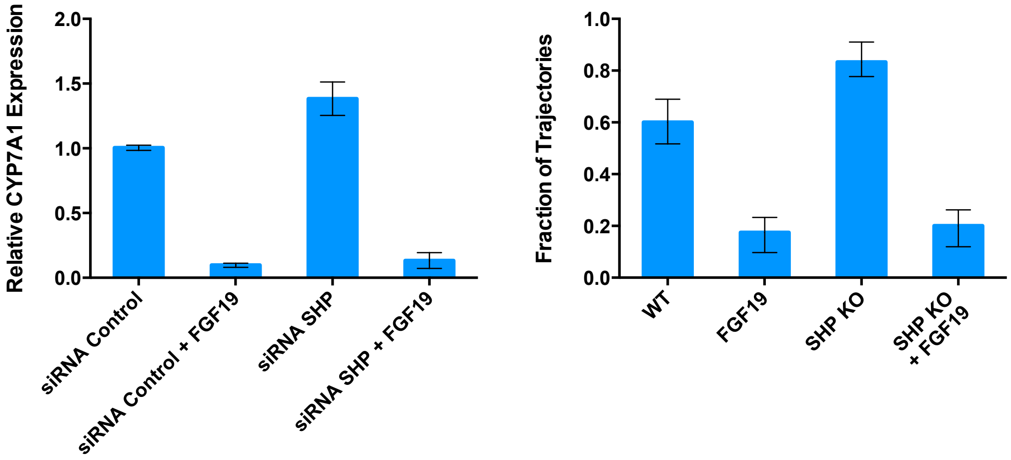

Supplementary Figure 4.7. Response of CYP7A1 transcript to SHP siRNA and FGF19 treatments. Experimental data from Figure 4C of Song *et al.* 2008 (left) and example token game trajectory (right). Both exhibit the same qualitative, burst behaviour.

Supplementary Table 4.7. Results obtained for CYP7A1 transcript (CYP7A1_mRNA) activation.

	FGF19 treatment	Control (WT)
Trajectories exhibiting CYP7A1 transcript activation behaviour	21	72
Fraction of trajectories	0.175	0.6
Lower CI	0.1174054	0.5105602
Upper CI	0.2527571	0.6832359

The number of trajectories exhibiting CYP7A1 activation is significantly lower in treated samples than in controls (the 95% CIs of treated system and control do not overlap). This is in agreement with experimental data (supplementary figure 4.7) where the CYP7A1 transcript is significantly lower in treated samples with respect to the control. Therefore, both experimental and simulation results fit the definition of 'decrease' behaviour.

4.2.8. SHP siRNA treatment increases relative CYP7A1 expression.

The simulation protocol described in section 4.2.7 was used to compare the number of trajectories in which CYP7A1 transcript exhibits activation behaviour. The SHP siRNA treatment was implemented as follows; the NR0B2_siRNA node was added to the network and connected by inhibitory edge to the SHP translation transition (NR0B2_Translation). An additional SHP transcript (NR0B2_mRNA) degradation transition was also added with a very high rate of 1000, which was connected by a read (activation) edge to NR0B2_siRNA. The NR0B2_siRNA node received one token after simulations ran for 1 arbitrary time unit, this resulted in inhibition of SHP translation and fast degradation of the SHP transcript.

Supplementary Table 4.8. Results obtained for CYP7A1 transcript (CYP7A1_mRNA) activation.

	SHP siRNA treatment	Control (WT)
Trajectories exhibiting CYP7A1 activation behaviour.	100	72
Fraction of trajectories	0.8333333	0.6
Lower CI	0.7565472	0.5105602
Upper CI	0.88944	0.6832359

The number of trajectories exhibiting CYP7A1 activation is significantly higher in the treated sample than control (the 95% CIs of treated system and control do not overlap). This is in agreement with experimental data (Supplementary Figure 4.7) where the CYP7A1 transcript is significantly higher in treated sample with relative to the control. Therefore, both experimental and simulation results fit the definition of 'increase' behaviour.

4.2.9. SHP siRNA & FGF19 treatment decreases relative CYP7A1 expression.

The simulation protocol described in section 4.2.7 was used to compare the number of trajectories in which CYP7A1 transcript exhibits activation behaviour. The FGF19 treatment described in section4.2.5 and SHP siRNA treatment described in section 4.2.8 were applied simultaneously.

Supplementary Table 4.9. Results obtained for CYP7A1 transcript (CYP7A1_mRNA) activation.

	SHP siRNA + FGF19 treatment	Control (WT)
Trajectories in which CYP7A1 transcript exhibits activation behaviour.	24	72
Fraction of trajectories	0.2	0.6
Lower CI	0.1382448	0.5105602
Upper CI	0.2803667	0.6832359

The number of trajectories exhibiting CYP7A1 activation is significantly lower in the treated sample than in the control (the 95% CIs of treated system and control do not overlap). This is in agreement with experimental data (supplementary figure 4.7) where the CYP7A1 transcript is significantly lower in treated sample relative to the control. Therefore, both experimental and simulation results fit the definition of 'decrease' behaviour.

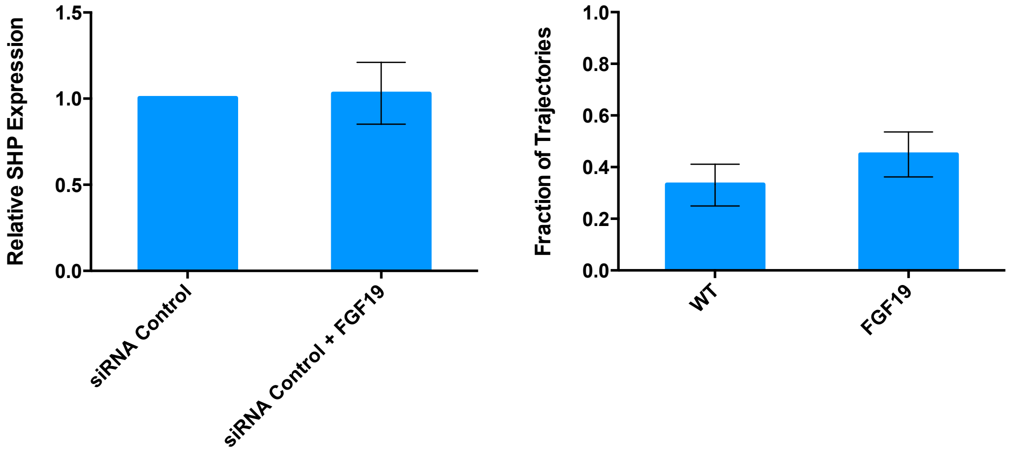

Supplementary Figure 4.8. Response of SHP transcript to control siRNA and FGF19 treatments. Experimental data from Figure 4B of Song *et al*. 2008 (left) and example token game trajectory (right). Both exhibit the same qualitative, burst behaviour.

4.2.10. SHP expression is not affected by FGF19.

Both experimental and simulation results shown in section 4.2.6 demonstrate that SHP transcript exhibits burst behaviour following FGF19 treatment. Here we used the same simulation as in section 4.2.7 to determine whether the number of trajectories in which SHP exhibits burst is significantly different in an FGF19 treated system than in the control.

Supplementary Table 4.10. Results obtained for SHP transcript (NR0B2_mRNA) burst.

	FGF19 treatment	Control (WT)
Number of trajectories in which SHP transcript exhibits burst behaviour.	54	40
Fraction of trajectories	0.45	0.3333333
Lower CI	0.3639174	0.2553174
Upper CI	0.5391846	0.421689

The 95% CI of treated and control system do overlap. Therefore, there is no significant difference between numbers of trajectories exhibiting burst behaviour in treated system and control. This is in agreement with experimental data (supplementary figure 4.10). Therefore, both simulation results and experimental data fit the definition of 'equal' behaviour.

4.2.11 Relative CYP7A1 expression increases after treatment with anti-FGF19 antibody

The simulation protocol described in section 4.2.7 was used to compare the number of trajectories exhibiting CYP7A1 activation in the GW4064 treated system and the system treated with GW4064 and anti-FGF19 antibody. The GW4064 treatment has been implemented as described in section 4.2.1. A Petri net node representing FGF19 antibody node was added to the system and connected by an inhibitory edge to the transition representing binding of FGF19 to its receptor (FGF1_activation). This mimics competitive inhibition of receptor activation by the antibody for FGF19 substrate. Following implementation of other perturbations, the FGF19 node received 1 token after 1 arbitrary unit of simulation time. Simulations were run for an additional 5.5 time units. Identical results were obtained when simulations were run for 6 and 10 time units after perturbation was applied.

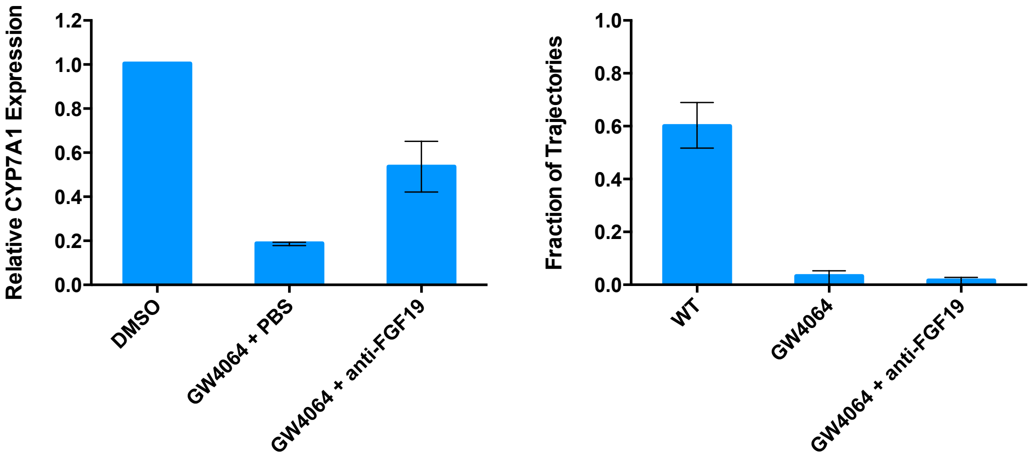

Supplementary Figure 4.9. Response of CYP7A1 transcript to anti-FGF19 antibody. Experimental data from Figure 6A of Song *et al*. 2008 (left) and example token game trajectory (right). Each exhibits different qualitative behaviours with respect to GW4064 controls.

Supplementary Table 4.11. Results obtained for CYP7A1 transcript (CYP7A1_mRNA) activation.

	**GW4064 + anti FGF19 antibody**	**Control (GW4064)**
Trajectories in which CYP7A1 transcript exhibits activation behaviour.	4	2
Fraction of trajectories	0.03333333	0.01666667
Lower CI	0.01303755	0.004582565
Upper CI	0.08258034	0.05873596

This simulation failed to reproduce the observed experimental behaviour. The experimental data (supplementary figure 4.9) demonstrate that the anti-FGF19 antibody inhibits downregulation of CYP7A1 expression with relative to GW4064 treated controls. In simulation results there is no significant difference between number of trajectories exhibiting CYP7A1 transcript activation behaviour in anti-FGF19 antibody and GW4064 treated systems. We could not improve this result by examining GW064 treated control system at different simulation timepoints. Therefore, we conclude that experimental data fit the definition of increase behaviour, while simulation results exhibit equal behaviour.

4.2.12. Relative CYP7A1 expression increases after treatment with FGFR4 siRNA

The simulation protocol described in section 4.2.7 was used to compare the number of trajectories exhibiting CYP7A1 activation in GW4064-treated systems and the system co-treated with GW4064 and FGFR4 siRNA. The GW4064 treatment was implemented as described in section 4.2.1. The FGFR4 siRNA treatment was implemented as follows, an FGFR4_siRNA node was added to the network and connected by an inhibitory edge to the FGFR4 translation transition (FGFR4_Translation). We also added an additional FGFR4 transcript (FGFR4_mRNA) degradation transition with a very high rate (1000), which was connected by a read (activation) edge to FGFR4_siRNA. Following implementation of other perturbations the FGFR4_siRNA node received 1 token after 1 arbitrary unit of simulation time. Simulations were run for an additional 5.5 time units. Identical conclusions were obtained when simulations were run for 6 and 10 time units after perturbation was applied

Supplementary Figure 4.10. Response of CYP7A1 transcript to FGFR4 siRNA. Experimental data from Figure 6D of Song *et al*. 2008 (left) and example token game trajectory (right). Each exhibits a different qualitative behaviours with respect to GW4064 controls.

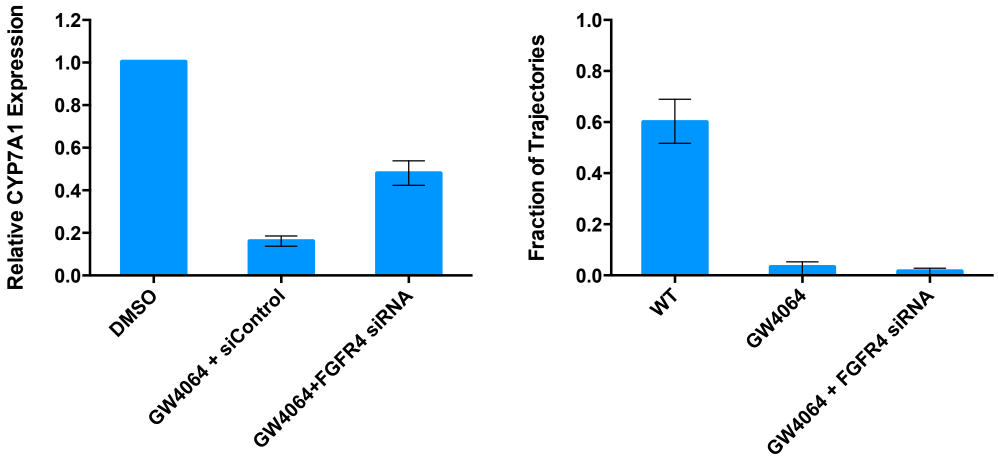

Supplementary Table 4.12. Results obtained for CYP7A1 transcript (CYP7A1_mRNA) activation.

	GW4064 + FGFR4 siRNA	Control (GW4064)
Trajectories in which CYP7A1 transcript exhibits activation behaviour.	4	2
Fraction of trajectories	0.03333333	0.01666667
Lower CI	0.01303755	0.004582565
Upper CI	0.08258034	0.05873596

This simulation failed to reproduce the observed experimental behaviour. The experimental data (supplementary figure 4.12) demonstrate that FGFR4 siRNA inhibits downregulation of CYP7A1 mRNA expression with respect to GW4064 control. In simulation results, there is no significant difference between the number of trajectories exhibiting CYP7A1 transcript activation behaviour in FGFR4 siRNA and GW4064 treated systems. We could not improve this result by examining GW064 treated control system at different simulation timepoints. Therefore, we conclude that experimental data fit the definition of increase behaviour, while simulation results exhibit equal behaviour.

4.2.13. Bile acid homeostasis

In sections 4.2.1-12 we evaluated the predictive power of the model by directly comparing simulation results with relative expression data collected from cultured human hepatocytes. In this section we test whether the model reproduces the known physiological behaviour of bile acid homeostasis (Li and Chiang, 2012; Chiang, 2009). Whole-organism, clinical studies are considerably more difficult than *in vitro* cell culture experiments and detailed experimental timecourses on relative amounts of bile acids as a function of perturbation in humans are not available. However, two basic properties of the physiological response to increased cholesterol load are well documented in the literature and reviewed in (Li and Chiang, 2012; Chiang, 2009). First, increased cholesterol in the portal blood results in an increase in bile acid production with cholesterol being the precursor of the primary bile acid synthesis pathways. Second, increased bile acid levels are a signal for downregulation of bile acid synthesis. High levels of bile acids are toxic and negative feedback loops prevent synthesis of harmful amounts of bile acid even if cholesterol levels remain high. We tested whether our model reproduces these two known properties of physiological response for the primary bile acids, chenodiol and cholate, produced by the hepatocyte.

We have monitored the metabolic flux towards chenodiol and cholate under conditions of constant, high cholesterol availability. The high cholesterol load has been simulated as a level of cholesterol that cannot be decreased (cleared) within the timescale of the simulation. Simulations started from an initial state, with 1 token present at each place representing an inactive gene and 0 tokens were assigned to all other places. This initial state represents the genotype of the system. Each trajectory of the QSSPN simulation was run in the following way. The simulation was run for 1 arbitrary time unit to allow all the genes to express their products under normal (physiological) conditions. Subsequently, the transition from “Cholesterol_source” to “Cholesterol_pool” was enabled, while all transitions consuming tokens from “Cholesterol_pool” were removed. This resulted cholesterol availability being high throughout the course of the simulation. Following application of this perturbation, the simulation was run for 10 arbitrary time units.
The objective nodes “Chenodiol_r” and “Cholate_r” monitored the value of chenodiol and cholate synthesis with 11 discrete activity levels (maximal level of ten tokens). Two properties of physiological response were tested. First, an increase in bile acid synthesis above the basal level in response to high cholesterol levels. Second, the return of bile acids to basal levels, despite the presence of high levels of cholesterol. Such as response replicates a negative feedback loop inhibiting excessive synthesis of bile acids the accumulation of toxic levels. Supplementary Figure 4.11 shows simulation trajectory exhibiting this behaviour. We used 3 tokens as the threshold between high and basal bile acid synthesis. Thus, physiological behaviour was assessed by the number of trajectories exhibiting burst behaviour, where basal level L_0 (definition in section 4.1) was defined as token status lower than 3.

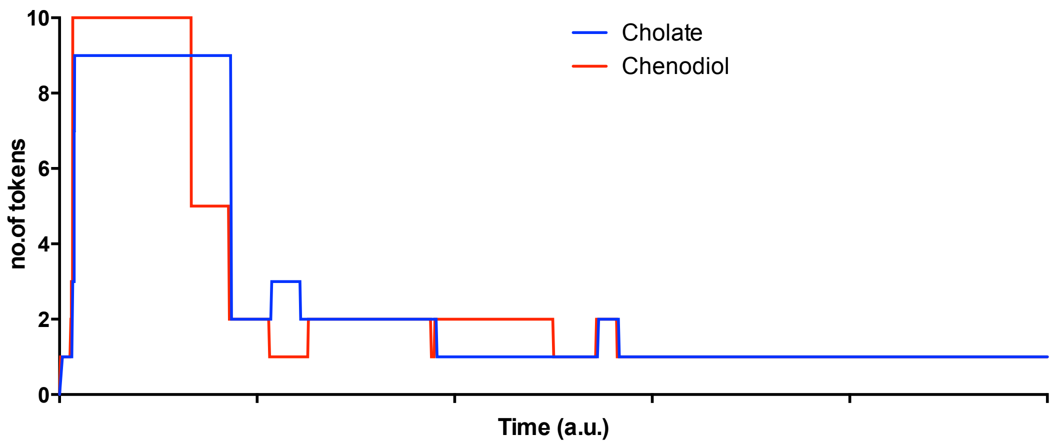

Supplementary Figure 4.11. Example trajectory showing response of Chenodiol and Cholate to permanent cholesterol rise at simulation time 0. Despite cholesterol levels in excess of that which could be cleared, bile acids returned to basal levels. This trajectory demonstrates prevention of excessive bile acid production through regulatory mechanisms in hepatocytes protecting against bile acid toxicity.

Supplementary Table 4.13. Results obtained for Chenodiol objective node (Chenodiol_r).

**Most Frequently Occurring Behaviour**	
	**Burst**
Number of trajectories	117
Fraction of trajectories	0.975
Lower CI	0.92907
Upper CI	0.9914618

Table 4.14. Results obtained for Cholate objective node (Cholate_r).

**Most Frequently Occurring Behaviour**	
	**Burst**
Number of trajectories	117
Fraction of trajectories	0.975
Lower CI	0.92907
Upper CI	0.9914618

We conclude that the cholate and chenodiol objective nodes both exhibit burst behaviour. This is consistent with literature knowledge on the physiological homeostatic response of bile acid synthesis to increased cholesterol. Increased cholesterol load results in transient increase of bile acid production, which is downregulated to prevent bile acid accumulation at toxic level.

4.3 Error function and predictive power of the model

4.3.1. Formulation of error function

In the previous sections we presented 14 simulations compared with experimental data on the relative molecular expression *in vitro* and known physiological behaviours of the system. Here we formulate an error function that uses results of the comparisons to quantitatively evaluate the predictive power of our model.

In our comparisons we classify experimental and simulation results to several classes of systems behaviour and compare whether observed and predicted behaviours are the same. The correlation between predicted and observed classes can be expressed using Matthews Correlation Coefficient (MCC), commonly used in the evaluation of machine learning algorithms (Baldi *et al.*, 2000). Following a well-established protocol, we first compile a confusion matrix for each of the 8 behaviours defined in Chapter 4.1. The Confusion Matrix calculated for behaviour, B, (e.g. inhibition) is shown in table 4.15.

Table 4.15. Confusion matrix as calculated for each behaviour, B

	**Simulation results exhibit behaviour B.**	**Simulation results do not exhibit behaviour B.**
**Experimental data exhibit behaviour B.**	TP_B	FN_B
**Experimental data do not exhibit behaviour B.**	FP_B	TN_B

Where: TP_B *(true positive)* – the number of comparisons in which experimental behaviour equals B and simulated behaviour equals B; TN_B *(true negative)* – the number of comparisons in which experimental behaviour is different than B and simulated behaviour is different than B; FP_B *(false positive)* – the number of comparisons in which experimental behaviour is different than B and simulated behaviour equals B; FN_B *(false negative)* – the number of comparisons in which experimental behaviour equals B and simulated behaviour is different than B.

In calculation of confusion matrices for behaviours activation, inhibition, burst, oscillation, constant, the results of the comparisons in sections 4.2.1-6 and 4.2.13 were used. Confusion matrices for increase, decrease, equal were calculated using the results of comparisons discusse in sections 4.2.7-12. This insured that the correct set of possible outcomes was used for the compilation of each matrix. The confusion matrices obtained for all 8 behaviours were subsequently added together resulting in total numbers of true positive (TP), true negative (TN), false positive (FP) and false negative (FN) results. The MCC was then calculated by the following formula:

$$MCC = \frac{TP \times TN + FP \times FN}{\sqrt{(TP+FP) \times (TP+FN) \times (TN+FP) \times (TN+FN)}}$$

MCC values are always between -1 and 1; -1 indicates total disagreement; 1 indicates total agreement. Completely random predictions would result in an MCC = 0, making MCC useful for evaluation of predictive power against a random baseline.

4.3.2. Predictive power of the hepatocyte model.

Supplementary Table 4.16 summarises evaluation of the predictive power of QSSPN simulations with hepatocyte model presented in Supplementary Figure 2.1 with parameters from Supplementary Table 3.1.

Supplementary Table 4.16. Comparison of behaviours exhibited by experimental data and simulation results.

Molecule	Experimental data	Experimental behaviour	Simulation behaviour
FGF19 transcript	Relative amount after treatment with GW4064 for 0, 0.6, 1, 3, 6, 24 hours (Section 4.2.1).	activation	activation
CYP7A1 transcript	Relative amount after treatment with GW4064 for 0, 0.6, 1, 3, 6, 24 hours (Section 4.2.2).	inhibition	inhibition
SHP transcript	Relative amount after treatment with GW4064 for 0, 0.6, 1, 3, 6, 24 hours (Section 4.2.3).	burst	burst
HNF4α transcript	Relative amount after treatment with GW4064 for 0, 0.6, 1, 3, 6, 24 hours (Section 4.2.4).	constant	constant
CYP7A1 transcript	Relative amount after treatment with FGF19 for 0, 0.6, 1, 3, 6, 24, 48 hours (Section 4.2.5).	inhibition	inhibition
SHP transcript	Relative amount after treatment with FGF19 for 0, 0.6, 1, 3, 6, 24, 48 hours (Section 4.2.6).	burst	burst
CYP7A1 transcript	Relative amount with respect to untreated control after treatment with FGF19 (Section 4.2.7)	decrease	decrease
CYP7A1 transcript	Relative amount with respect to untreated control after treatment with SHP siRNA (Section 4.2.8)	increase	increase
CYP7A1 transcript	Relative amount with respect to untreated control after treatment with SHP siRNA and FGF19 (Section 4.2.9)	decrease	decrease
SHP transcript	Relative amount with respect to untreated control after treatment with FGF19 (Section 4.2.10)	equal	equal
CYP7A1 transcript	Relative amount with respect to GW4064 treatment after FGF19 antibody treatment (Section 4.2.11)	increase	equal
CYP7A1 transcript	Relative amount with respect to GW4064 treatment after FGFR4 siRNA (Section 4.2.12)	increase	equal
Chenodiol	Physiological response to rise of cholesterol. (Section 4.2.13)	burst	burst
Cholate	Physiological response to rise of cholesterol. (Section 4.2.13)	burst	burst

Number of True Positive predictions (TP)	12
Number of True Negative predictions (TN)	42
Number of False Positive predictions (FP)	2
Number of False Negative predictions (FN)	2
**Matthews Correlation Coefficient (MCC)**	**0.812**

The TP, TN, FP, FN values are calculated by adding 8 confusion tables calculated for all 8 behaviours (see section 4.3). For example, 2 false positives come from confusion table for behaviour "equal", where this behaviour was falsely predicted in sections 4.11 and 4.12. False negative results come from confusion table of increase behaviour, which was falsely rejected in sections 4.11 and 4.12. All other predictions were true. In summary, the experimentally observed behaviour was correctly identified in 12/14 (86%) of comparisons. Alternatively, MCC may be used as a more sophisticated measure of error. The MCC value of 0.812 indicates that predictions are far superior to a random prediction of outcomes, but that perfect agreement with experimental data has not been achieved.

5 THE CHOICE OF QUALITATIVE PARAMETERS.

Supplementary Table 3.1 shows the set of qualitative parameters for which our model achieves very good predictive power (MCC = 0.812) as evaluated by direct comparison of simulation results with experimental data (Section 4). Here, we discuss the choice of the parameters and present results of additional simulations that show the robustness of qualitative results to substantial change of parameter values. We argue that QSSPN parameters are indeed qualitative thresholds, reflective of qualitative knowledge of the system and that exact quantitative parameter values do not need to be known or fitted.

The parameters c_1, N, $\mu_1(x)$, $\mu_2(x)$ are default parameters, which are set automatically according to network connectivity alone. Every place has a maximal number of tokens equal to N and its state is defined by an integer number of tokens in a range of [0, N]. Each transition is by default set to be stochastic with a rate constant of 1. Activity table $\mu_1(x)$ is used to implement basic Petri net (PN) definitions of edge and read edge: if the state of pre-place is 0, the propensity of the transition is 0; if there are more than 0 tokens, the propensity of transition is greater than 0 and the transition can fire (the only difference between an edge and a read edge is that the token is not consumed from a pre-place connected by a read edge on transition firing) . Likewise, $\mu_2(x)$ was used to implement definition of inhibitory edge: if the pre-place has more than 0 tokens, the propensity of the transition is multiplied by 0 and the transition cannot fire. When the network is constructed, each place, transition and edge is assigned these default parameters, which are used in the simulation unless changed later during the modelling process. The majority of places (189/201) and edges (441/481) in our hepatocyte model were assigned these default parameters. However, only 98/210 transitions were set to default, since many transitions in our general gene expression model (see below) were set as immediate.

The general QSSPN parameters represent the abstraction level of our model rather than parameters, which are adjusted to reproduce experimental data. The maximal number of tokens N is set to 2 reflecting our discretisation of molecular activity. We are interested in basal (0 tokens), transient (1 token) and fully active (2 tokens) states of the molecules in the system. We are not attempting to represent molecular activities at any more resolution, since we do not have quantitative data on molecular concentrations. However, available timecourses of relative molecular activity let us distinguish, not only the basal and fully activate state, but also the transient state, where molecular activity increases or decreases. Any change in parameter N would completely change our abstraction level and require changes to multiple parts of the model (e.g. threshold values in all activity tables would have to be re-evaluated).

In our qualitative simulations, we use time exclusively to order the sequence of transitions and do not interpret it as a physical time. The c_1 represents a default rate of 1 transition firing per 1 arbitrary time unit, with all other rates set as faster or slower than the default. The change of c_1 would be equivalent to re-scaling the system. All rates would have to be multiplied by the same factor and maximal simulation time would have to multiplied by the reciprocal of this factor; qualitative result would not change.

The parameters of gene expression process have been selected in such a way that meaningful changes in gene expression were observed within the simulation time of 10 arbitrary units. Again, we want to stress that any other arbitrary maximal simulation time length could have been chosen to evaluate qualitative behaviours, following simple rescaling of all rate constants. The choice of the maximal simulation time of 10 arbitrary units lead us to setting promoter activation rate (c_2) to be 100 times faster than default rate and the promoter and binding site inactivation rates (c_5) to be 10 times faster than default rate. We assume that formation of a transcriptionally active gene complex is rate limiting and use immediate transcription and translation transitions. We further assume that the dissociation of the stable complex between transcription factor and binding site is slower than promoter activation, otherwise the genes could not be activated. Finally, we state that transcript and protein degradation processes are slower than transcription regulatory events and we use default rate of 1. For all genes apart of SHP (see below) it was not necessary to adjust the default values of degradation parameters. The above assumptions reflect the basic qualitative knowledge of gene expression process and we note that the model using these assumptions is capable of predicting qualitative dynamic behaviours as demonstrated by comparison with experimental data.

To demonstrate robustness of QSSPN results to perturbation of exact, quantitative values of the parameters we have performed additional simulations where c_5 and c_2 values were decreased twofold. Supplementary Table 5.1 shows that none of the qualitative behaviours changed in the simulation where c_2 was set to 50 instead of 100, while keeping all other model settings unchanged and that none of the qualitative behaviours have changed when c_5 was changed from 10 to 5. As discussed above these two parameters are key to set relative timescales of gene activation and inactivation with respect to other processes. The fact that perturbation of these two key parameters does not make any difference to results demonstrates that QSSPN parameters are indeed qualitative rather than quantitative. The parameters are used to express qualitative knowledge about which molecular processes are faster than others, rather than to represent quantitative rate constants, which are not generally available.

During modelling process we have discovered that behaviour of SHP gene can be correctly reproduced only if we assume that SHP protein is more stable than other proteins in the system and that inhibition of SHP promoter by SHP protein is not complete. To introduce these assumptions we used parameters c_{10}, c_{11} and $\mu_5(x)$. While these parameters were adjusted to reproduce experimental data, we did not have to perform fitting of their quantitative values. Simple setting of the order of magnitude of these parameters was sufficient to improve predictive power of the model. Moreover, as discussed in the Main Manuscript, the stability of SHP protein is corroborated by an experimental publication that was not used during the original modelling process. Importantly, this shows that qualitative modelling with QSSPN is capable of providing novel mechanistic insight into molecular processes in human cells which may later be confirmed through laboratory

experimentation. In the following section we also show that alternative qualitative modelling methods are in principle not able to correctly reproduce SHP behaviour.

Supplementary Table 5.1. Sensitivity of QSSPN simulation to changes in qualitative rate parameters relative to c_1.

Molecule	Experimental data	Experimental behaviour	Original model	c_2=50	c_5=5
FGF19 transcript	Relative amount after treatment with GW4064 for 0, 0.6, 1, 3, 6, 24 hours (Comparison 1).	activation	activation	activation	activation
CYP7A1 transcript	Relative amount after treatment with GW4064 for 0, 0.6, 1, 3, 6, 24 hours (Comparison 2).	inhibition	inhibition	inhibition	inhibition
SHP transcript	Relative amount after treatment with GW4064 for 0, 0.6, 1, 3, 6, 24 hours (Comparison 3).	burst	burst	burst	burst
HNF4a transcript	Relative amount after treatment with GW4064 for 0, 0.6, 1, 3, 6, 24 hours (Comparison 4).	constant	constant	constant	constant
CYP7A1 transcript	Relative amount after treatment with FGF19 for 0, 0.6, 1, 3, 6, 24, 48 hours (Comparison 5).	inhibition	inhibition	inhibition	inhibition
SHP transcript	Relative amount after treatment with FGF19 for 0, 0.6, 1, 3, 6, 24, 48 hours (Comparison 6).	burst	burst	burst	burst
CYP7A1 transcript	Relative amount with respect to untreated control after treatment with FGF19 (Comparison 7)	decrease	decrease	decrease	decrease
CYP7A1 transcript	Relative amount with respect to untreated control after treatment with SHP siRNA (Comparison 8)	increase	increase	increase	increase
CYP7A1 transcript	Relative amount with respect to untreated control after treatment with SHP siRNA and FGF19 (Comparison 9)	decrease	decrease	decrease	decrease
SHP transcript	Relative amount with respect to untreated control after treatment with FGF19 (Comparison 10)	equal	equal	equal	equal
CYP7A1 transcript	Relative amount with respect to GW4064 treatment after FGF19 antibody treatment (Comparison 11)	increase	equal	equal	equal
CYP7A1 transcript	Relative amount with respect to GW4064 treatment after FGFR4 siRNA (Comparison 12)	increase	equal	equal	equal
Chenodiol	Physiological response to rise of cholesterol. (Comparison 13)	burst	burst	burst	burst
Cholate	Physiological response to rise of cholesterol. (Comparison 14)	burst	burst	burst	burst
Number of correct predictions			12	12	12
**Matthews Correlation Coefficient**			**0.812**	**0.812**	**0.812**

We conclude that QSSPN is a qualitative modelling method, which does not require knowledge or fitting of quantitative values of molecular concentrations and interaction rate constants. Qualitative parameters of the model are predominantly used to formulate rules expressing knowledge about molecular network connectivity. However, existing knowledge about some classes of molecular processes being faster or slower than others can also be incorporated without the need of detailed quantitative measurements or fitting of exact quantitative parameter values. In Section 4 we show that QSSPN modelling approach achieves very good results in predicting qualitative dynamic behaviours in clinically relevant human cell system and in section 7 we show that it outperforms alternative qualitative modelling approaches.

6 DYNAMIC BEHAVIOURS IN GENE KNOCKOUT SIMULATIONS

Supplementary Table 6.1. Definition of dynamic behaviours monitored in computational gene knockout simulations (Fig 5).

Dynamic behaviour	Definition	Interpretation
'Slow cholesterol clearance'	Rise of the 'cholesterol_pool' node token status above basal level of 2 and subsequent return to a basal level at a time later than 3 arbitrary time units.	Disruption to the maximal efficiency of cholesterol clearance i.e. inhibition of either cholesterol conversion to BAs or inhibition of cholesterol transport.
'long cholate burst'	Objective node 'Cholate_r' rising to the level of more than 2 tokens for more than 3 time units.	Elevated synthesis of BAs is a major disruption of homeostatic function in our system. Disruption of cholate homeostasis was monitored as the prolonged activation of cholate biosynthetic flux above the basal level.
'long chenodiol burst'	Objective node 'Chenodiol_r' rising to the level of more than 2 tokens for more than 3 time units.	Elevated synthesis of BAs is a major disruption of homeostatic function in our system. Disruption of chenodiol homeostasis was monitored as the prolonged activation of chenodiol biosynthetic flux above the basal level.
'FGF19 protein burst'	The node FGF19 rising to the level of 2 tokens for more than 0.01 time units.	Secretion of FGF19 cytokine.
'MAPK pathway activation'	Permanent rise of MAPK kinase output node, cFOS, to the level of 2 tokens.	Activation of MAPK signalling cascade.
'CYP7A1 mRNA burst'	CYP7A1_mRNA node to the level of 2 tokens for more than 0.01 time units	Transient rise of CYP7A1 mRNA level.

7 COMPARISON OF QSSPN WITH EXISTING APPROACHES

To the best of our knowledge, the modelling of bile acid homeostasis in the human hepatocyte presented herein, is the first quasi-steady state simulation integrating gene regulation in human cells with a genome-scale metabolic network. Below, we argue that regulatory FBA (rFBA) is the only modelling approach other than QSSPN, which, in principle, is applicable to the dynamic simulation of a large-scale regulatory network coupled to whole cell metabolism in human cells. We have constructed an rFBA version of our test case model and directly compared QSSPN and rFBA. This comparison shows that QSSPN achieves greater predictive power than rFBA when applied to the simulation of the benchmark experiments presented in section 4.2.

There are following published, quasi-steady state approaches that integrate FBA of genome scale metabolic networks (GSMN) with dynamic simulation of slower processes: **dFBA** (Varma and Palsson, 1994), **rFBA** (M. W. Covert *et al.*, 2001), **iFBA** (M. W. Covert *et al.*, 2008), **idFBA** (Min Lee *et al.*, 2008), "**diverse mathematics**" (Karr *et al.*, 2012). In **dFBA** the nutrient consumption and secretion fluxes calculated by FBA are integrated into Ordinary Differential Equation (ODE) models of extracellular metabolite concentrations. While the simulation is dynamic and based on the quasi-steady state assumption, it is not applicable to our test case since it does not allow modelling of dynamic processes within the cell and so gene regulation cannot be modelled. A modified version of dFBA has recently been used to couple physiologically-based pharmacokinetic models (PBPK) of ammonia and paracetamol to HepatoNet1 model of human hepatocyte metabolism (Krauss *et al.*, 2012). We would highlight however, that this model did not contain any gene regulatory network or other intracellular regulatory processes. Instead, a steady state FBA simulation has been coupled to a dynamic model of selected extracellular substances within physiological compartments.

The **rFBA** method integrates, qualitative, Boolean models of gene regulation with FBA of whole-cell metabolism through quasi-steady state assumption. This approach does not allow representation of our test case model at the same level of detail. The rFBA approach represents whole genes as variables and specifies gene interaction rules, while in QSSPN we represent DNA, mRNA, protein and other small molecule species as separate variables. However, rFBA can, in principle, be used to simulate a coarse-grained version of our model and generate results, which are comparable to benchmark data sets (Section 4.2). In section7.1, we discuss rFBA in greater detail and demonstrate why it cannot be applied to the human cell without algorithm modifications. Subsequently we evaluate the performance of the adjusted rFBA method in predicting the results of experimental data and compare its predictive power with QSSPN.

The rFBA method has been further extended by **iFBA** (M. W. Covert *et al.*, 2008). The rFBA simulation including metabolic model and Boolean gene regulatory network is coupled with ODE simulations of dynamic processes for which quantitative parameters are available. These processes include parts of gene regulatory networks for which detailed kinetic models are available. The iFBA approach has been applied to the simulation of diauxic shift in *E. coli*, where kinetic models of PTS transport and catabolic regulation have been parameterised after years of focussed experimental effort (Bettenbrock *et al.*, 2006). To our knowledge there are no kinetic data of sufficient quality to parameterise many parts of our model of bile acid synthesis regulation in human hepatocytes. This makes our model a good, general test case study as detailed, robust kinetic parameters are commonly unavailable for molecular systems of clinical interest. Therefore, applying the iFBA method to our test case would be equivalent to using rFBA, as no ODE model would be included. The **idFBA** (Min Lee *et al.*, 2008) is another method where ODE simulation is coupled to FBA and so the same arguments apply. The idFBA has been tested on a very well studied microbial system and parameterisation of the ODE component of the model would not be possible for our test case.

Recently, the "**diverse mathematics**" approach has been applied to integrate models of molecular processes expressed in a wide range of modelling frameworks to perform detailed dynamic simulations of *Mycoplasma genitalium* (Karr *et al.*, 2012). The modelling frameworks were linked through shared variables and did include a very small, but still whole-cell metabolic model simulated with FBA and integrated with the remaining part of the model in quasi-steady state framework. The level of detail achieved in this work has only been possible because of the availability of experimental data resulting from unprecedented, coordinated experimental efforts focussed on this minimal genome bacterium (Kuhner *et al.*, 2009; Yus *et al.*, 2009; Guell *et al.*, 2009). Experimental studies on human cells are very far away from this level of detail and thus the "diverse mathematics" approach cannot be applied.

Another approach to combining FBA of whole-cell metabolism and qualitative models of large-scale gene regulatory networks is construction of constraint-based models that analyse steady states feasible in the integrated system. The SR-FBA (Shlomi *et al.*, 2007) is a pioneering method in this field and PROM is an important contribution where gene regulatory network rules are automatically derived from transcriptome data using probabilistic approaches (Chandrasekaran and Price, 2010). By definition, these methods are not applicable to our test study as they do not perform dynamic simulation. The dynamic behaviours of burst, oscillation, activation and inhibition analysed in our benchmark simulations and genotype-phenotype relationship study, cannot be obtained in simulations that exclusively analyse steady states of an integrated gene regulatory/metabolic system. The SR-FBA and related approaches cannot be applied to dynamic simulations of bile acid homeostasis in human hepatocytes. We would also like to note that the coupling of human metabolism to circadian clocks and other dynamic processes is now very well documented (Li and Chiang, 2012). Regulatory processes are of a fundamentally dynamic nature, and thus the analysis of steady states alone is likely to have a limited application in analysis of the large scale molecular interaction networks within the human cell.

Finally, in the Discussion chapter of main manuscript we mention two approaches, which motivated development of QSSPN. In the Signalling PN approach, Monte Carlo simulation of token game trajectories has been used for qualitative modelling of signalling networks alone. The calculation of propensity function in QSSPN (Equations 1,2 in main manuscript) is strongly motivated by a long standing effort of De Jong's group (Batt *et al.*, 2012; Baldazzi *et al.*, 2010), where piecewise linear equations are used to construct a qualitative, coarse-grained

equivalent of ODE-based dynamic system models. We would like to stress that none of these methods has ever been coupled to FBA simulations to perform quasi-steady state simulation of regulatory network integrated with whole-cell scale metabolic model. Thus, these methods cannot be applied to simulation of our model.

We conclude that rFBA is the only previously published approach that can be used to model our test case system and simulate results comparable with benchmark experimental datasets (Chapter 4.2). In the following chapter we will present direct comparison between QSSPN and rFBA.

7.1 Comparison of QSSPN with Regulatory FBA (rFBA).

The rFBA algorithm was formulated in 2001 by Covert, Schilling and Palsson (M. W. Covert *et al.*, 2001) and was applied to simulating catabolite repression and diauxic shifts in bacterial batch cultures. While detailed formulation of the method is available in the original publication and will not be re-iterated here, we discuss some key assumptions of the rFBA algorithm, which make its application simulating of human cells very challenging. First, the original rFBA algorithm requires formulation of the 'biomass' reaction and interpretation of biomass flux as a growth rate in cell culture. During the simulation, timecourses of extracellular metabolite concentrations and cellular biomass are generated, using the dFBA method (Varma and Palsson, 1994). Thus, in every timestep of the simulation, the biomass synthesis rate is maximised. The maximal growth rate and extracellular metabolite uptake/secretion fluxes obtained in the FBA solution are then incorporated into the coupled ODEs, which update both the biomass in the culture and the amount of extracellular metabolites in the media. The ODE model takes into account the increase in nutrient consumption/release due to growing number of cells (biomass) that consume/release nutrients. It is also important to note that the transport flux values used by the method are non-unique; they are extracted from one of many possible flux distributions that sustain a unique, maximal growth rate.
The gene regulatory network is included as a set of Boolean rules that determine the states of Boolean variables representing regulatory genes and genes encoding metabolic enzymes. One Boolean variable represents a gene; the model does not resolve transcripts and proteins. The state of regulatory genes depends on outputs of the metabolic reaction network and the bounds of reactions in the metabolic model depend on the state of metabolic genes. Again, information about the availability of metabolites interacting with transcription factors (e.g. lactose to determine state of lactose operon) is formulated in terms of thresholds of fluxes obtained in a non-unique FBA solution corresponding to maximal growth rate. Iteration of dFBA and Boolean rules result in simulation timecourses of extracellular metabolites that correctly reproduce metabolic shifts through activation/repression of metabolic operons (e.g. lactose starts to be consumed only after glucose is depleted). To correctly reproduce the lag times in metabolic shifts, Covert and colleagues introduced a single delay time for protein synthesis and degradation. The state update of Boolean variables is delayed with respect to time when Boolean rules are evaluated.

The major limitation of the rFBA method in simulating human cells is its reliance on the maximisation of biomass flux and interpretation of this flux as a growth rate of cellular culture in logarithmic growth phase. In our test case study, both hepatocytes cultured *in vitro* and *in vivo* hepatocytes do not divide within the same timescales as the behaviours of interest (e,g bile acid homeostasis) occur. Even if metabolic requirements of the cell were summarised in some form of 'biomass' equation, its interpretation as a cellular growth rate would be wrong: biomass of liver cells does not logarithmically increase in *in vitro* culture experiments and in the liver organ. For this reason, the HepatoNet1 model does not contain any biomass formula (Gille *et al.*, 2010). The authors use FBA optimisation solely to test the feasibility of metabolic functions; growth rate is never simulated. Likewise, Krauss and colleagues had to modify the original dFBA (dFBA alone, rather than rFBA, no gene regulation included) method to couple the HepatoNet1 to PBPK model of extracellular substance dynamics (Krauss *et al.*, 2012). First, they did not calculate biomass growth and did not include biomass into calculating consumption rates. Second, they used 'case specific objectives' to obtain flux distributions for particular sets of boundary conditions.

Due to the dependence of rFBA method on cellular growth simulation, we are unaware of any existing software implementing rFBA that can be used for simulation of our benchmark case studies. The software used in the original work of Covert, Shilling and Palsson (M. W. Covert *et al.*, 2001) has not been published. The COBRA software toolbox (Schellenberger *et al.*, 2011) implements rFBA in 'dynamicRFBA' function. We examined the MATLAB code of this function and it does indeed rely on such a biomass growth assumption. Moreover, the dynamicRFBA function does not implement a delay time in the evaluation of gene states. The MATLAB code implementing rFBA is also available in the supplementary material of the iFBA publication (M. W. Covert *et al.*, 2008). Again, it implements microbial culture simulation and is not applicable to the human cell system where there is no net cell division within the timescale of simulations.

We conclude that to apply rFBA to benchmark experimental datasets and compare it with QSSPN, the original method needs to be modified to remove the dependence on the growth rate calculation. We have formulated a coarse-grained version of our hepatocyte model with the regulatory network modelled using Boolean rules. We implement this modified version of rFBA with three different parameterisations of delay times and compare rFBA with QSSPN.

7.1.1. The rFBA model of bile acid homeostasis in human hepatocytes.

We have represented the dynamic transition part of our model (Supplementary Figure 2.1, Supplementary Table 3.1) using the coarse-grained format of a Boolean gene regulatory network. Thus, each gene is represented by one Boolean variable for which a FALSE or TRUE state was determined by evaluation of the rule involving other Boolean variables and threshold conditions. These are dependant on the values of objective functions monitoring the availability of metabolites interacting with transcription factors. The bounds of HepatoNet1 (Gille *et al.*, 2010) reactions, which required products of genes included in regulatory network, were set to basal or fully active level

for FALSE and TRUE states of regulatory network, respectively. During model construction, we represented exactly the same set of molecular interactions as in our QSSPN model. We also used the same mapping of HepatoNet1 reactions to metabolic genes, and the same definition of objective functions and thresholds to monitor metabolites interacting with transcription factors.

Supplementary Table 7.1. The rFBA version of human hepatocyte model.

Gene	Type of Product	Boolean rule
ABCG5_G8	Enzyme (1 HepatoNet1 flux)	NR1H3 AND NR2B1
ABCA1	Enzyme (17 HepatoNet1 fluxes)	NR1H3 AND NR2B1
UGT2B4	Enzyme (1 HepatoNet1 flux)	(NR1H3 AND NR2B1) OR (NR2B1 AND NR1H4)
SLC27A5	Enzyme (7 HepatoNet1 fluxes)	NR2B1 AND NR1H4
ABCB4	Enzyme (4 HepatoNet1 fluxes)	NR2B1 AND NR1H4
BAAT	Enzyme (3 HepatoNet1 fluxes)	NR2B1 AND NR1H4
ABCC2	Enzyme (3 HepatoNet1 fluxes)	NR2B1 AND NR1H4
ABCB11	Enzyme (4 HepatoNet1 fluxes)	NR2B1 AND NR1H4
CYP27A1	Enzyme (3 HepatoNet1 fluxes)	NR5A2 AND HNF4A AND (NOT NR0B2) AND (NOT cFOS)
CYP8B1	Enzyme (1 HepatoNet1 flux)	NR5A2 AND HNF4A AND (NOT NR0B2) AND (NOT cFOS)
CYP7A1	Enzyme (1 HepatoNet1 flux)	NR5A2 AND HNF4A AND (NOT NR0B2) AND (NOT cFOS)
SLC10A1	Enzyme (11 HepatoNet1 fluxes)	NR5A2 AND HNF4A AND (NOT NR0B2) AND (NOT cFOS)
SLCO1B1	Enzyme (95 HepatoNet1 fluxes)	NR5A2 AND HNF4A AND (NOT NR0B2) AND (NOT cFOS)
SLCO1B3	Enzyme (23 HepatoNet1 fluxes)	NR5A2 AND HNF4A AND (NOT NR0B2) AND (NOT cFOS)
SLCO1A2	Enzyme (94 HepatoNet1 fluxes)	NR5A2 AND HNF4A AND (NOT NR0B2) AND (NOT cFOS)
NR1H3	Transcription factor	Objective(Hydroxycholesterol) > 3
NR2B1	Transcription factor	TRUE
NR1H4	Transcription factor	*Objective(Chenodiol_Intracellular) > 3 OR Objective(Cholate_Intracellular) > 3
FGF19	Cytokine	NR2B1 AND NR1H4
NR0B2	Regulatory protein	((NR1H3 AND NR2B1) OR (NR2B1 AND NR1H4)) AND (NOT NR0B2)
NR5A2	Transcription factor	TRUE
HNF4A	Transcription factor	TRUE
cFOS	Transcription factor	FGF19 AND FGFR4
FGFR4	Receptor	TRUE

*Objective(name) denotes the value of FBA maximisation of objective function indicated by "name" on HepetoNet1 model with physiological import physiological export PIPES boundary conditions.

Supplementary Table 7.1 shows the Boolean rules of the regulatory network coupled to FBA simulation of HepatoNet1 (Gille *et al.*, 2010). The model consists of 24 genes represented by Boolean variables. The state of each variable is calculated using Boolean expression rules involving other variables and threshold conditions on objective function values. The variables representing genes encoding enzymes are linked to fluxes in HepatoNet1. The simulations proceed through discrete timesteps. We used the number of timesteps as an arbitrary time unit. At each timestep, the state of all Boolean variables is updated. Each variable is assigned a state resulting from evaluation of the Boolean rules for states of other variables at previous timestep. Following the state update for genes, the flux bounds in HepatoNet1 are updated and objective functions featuring in Boolean rules are re-evaluated. The simulation is deterministic, producing a single simulation trajectory.

We have implemented three variants of rFBA simulation that incorporate delayed state changes of Boolean variables in three different ways. In **rFBA1** there is no delay in variable state change, all variables are updated as described above. This corresponds to rFBA protocol as implemented in the COBRA Toolbox (Schellenberger *et al.*, 2011).
In **rFBA2** we follow the original rFBA algorithm (M. W. Covert *et al.*, 2001) and implement a single delay T_d time for the state change of Boolean variables. Thus, if the state of a Boolean variable is FALSE and its rule evaluates as TRUE, the state will be changed to TRUE only after T_d timesteps. No changes to the state of the system occurring within delay time can alter the state change committed to at the beginning of delay time period. In our simulations we used $T_d = 10$ timesteps.
The **rFBA3** approach is our own, novel, variant of the rFBA algorithm, which introduces separate activation and decay delay times specific to each gene. If the state of Boolean variable i is FALSE and its rule evaluates as TRUE, the state of this variable will be changed after activation delay time $T_{a,i}$ timesteps. If the state of Boolean variable is TRUE and its rule evaluates as FALSE, the state change will take place after decay time of $T_{d,i}$ timesteps. The delay times used in the three rFBA methods are summarised in Supplementary Table 7.2 below.

Supplementary Table 7.2. Delay times used in rFBA simulations.

Gene	rFBA1		rFBA2*		rFBA3*	
	Activation delay	Decay delay	Activation delay	Decay delay	Activation delay	Decay delay
ABCG5_G8	0	0	10	10	0	10
ABCA1	0	0	10	10	0	10
UGT2B4	0	0	10	10	0	10
SLC27A5	0	0	10	10	0	10
ABCB4	0	0	10	10	0	10
BAAT	0	0	10	10	0	10
ABCC2	0	0	10	10	0	10
ABCB11	0	0	10	10	0	10
CYP27A1	0	0	10	10	0	10
CYP8B1	0	0	10	10	0	10
CYP7A1	0	0	10	10	0	10
SLC10A1	0	0	10	10	0	10
SLCO1B1	0	0	10	10	0	10
SLCO1B3	0	0	10	10	0	10
SLCO1A2	0	0	10	10	0	10
NR1H3	0	0	0	0	0	0
NR2B1	0	0	0	0	0	0
NR1H4	0	0	0	0	0	0
FGF19	0	0	10	10	0	80
NR0B2	0	0	10	10	0	50
NR5A2	0	0	10	10	0	0
HNF4A	0	0	10	10	0	0
cFOS	0	0	10	10	50	50
FGFR4	0	0	10	10	0	0

* Delays model time difference between gene activation/inactivation and gene product appearance/disappearance due to slow protein synthesis/degradation processes. The genes, which were assumed to be constitutively expressed were not delayed, as there was no net state change due to protein/mRNA synthesis degradation. This also concerns genes which state was changed exclusively by results of metabolite objective functions. Activity of these genes was changed at the level of ligand binding to protein, rather than by protein synthesis/degradation.

7.1.2. Implementation of rFBA methods.

It has been shown previously, (Steggles *et al.*, 2007) that PNs can be used to implement Boolean gene regulatory networks (Note: gene regulatory network alone; integration with FBA has not been considered). Here, we used PNs to implement the Boolean gene regulatory network described in Supplementary Table 7.1 and subsequently used our QSSPN software to integrate it with the HepatoNet1 GSMN. This not only simplified implementation of rFBA methods, but also allowed use of the same objective function definitions as in QSSPN simulations, facilitating direct comparison of both methods. Our PN implementation of a Boolean network differs from that of Steggles and colleagues for two reasons. Firstly, we implemented a delayed Boolean network; previous work did not consider delays. Secondly, we used instantaneous transitions to implement synchronous updates; Steggles *et al* used exclusively stochastic transitions and implemented synchronisation with additional PN circuitry. Below we outline our PN implementation of a gene (Boolean variable) used in the subsequent rFBA models using CYP8B1 as an example (supplementary figure 7.1).

The Boolean variable representing a gene is implemented with a PN place with the maximal number of tokens set to 1. This place can assume only two states: 0 and 1 representing FALSE and TRUE states of the Boolean variable, respectively. The places CYP8B1, cFOS, HNF4A and NR0B2 shown on the figure implement Boolean variables. The CYP8B1_rule transition evaluates the Boolean rule; it fires only when pre-places connected by read edges have 1 token and pre-places connected by inhibitory edges have 0 tokens. Therefore, this transition fires only when the rule: NR5A2 AND HNF4A AND (NOT NR0B2) AND (NOT cFOS) is true. When this transition is enabled the place CYP8B1_ON receives one token. In every subsequent transition in which CYP8B1_rule can fire, this place receives one token and loses one token, thus its state remains 1. When CYP8B1_rule cannot fire, the token is removed and the state of CYP8B1_ON returns to 0. The rules involving OR statements are implemented by multiple transitions evaluating AND and NOT terms. Multiple transitions can still only raise the number of CYP8B1_ON tokens to 1 (maximal number of tokens bound), so the node will return to 0 as soon as none of the multiple transitions can fire (OR condition).

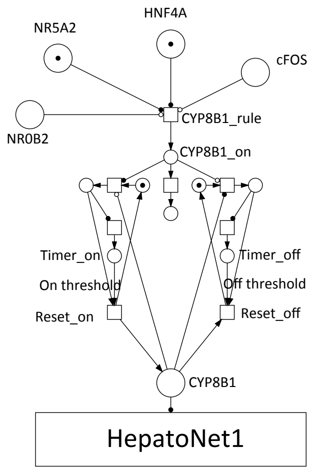

Supplementary Figure 7.1. Petri Net implementation of delayed Boolean variable representing a gene, CYP8B1, in rFBA simulations.

If the state of CYP8B1_ON is 1, the activation delay timer commences. The node Timer_on accumulates tokens until the number of tokens reaches the threshold, specified by the On_threshold read edge, which activates Reset_on transition. This transition places one token on CYP8B1, thus setting the state of the Boolean variable to TRUE and resets the activation timer. If CYP8B1 is TRUE the decay timer commences, when the CYP8B1_rule cannot fire. This timer will set CYP8B1 to 0 after the Timer_off node accumulates the number of tokens exceeding the Off_threshold. Both timers are mutually exclusive. All transitions except Reset_on and Reset_off are instantaneous; they fire synchronously and the number of tokens on Timer_on/Timer_off nodes is equal to the number of timesteps since the timer was activated. The Reset_on and Reset_off transitions are fast transitions with very high rates (1000), as such they consume the maximal numbers of tokens from every node and reset Timer nodes to 0. To summarise, PN shown on Supplementary Figure 7.1 implements Boolean variable with separate delays for changing to TRUE and FALSE state as defined in Chapter 7.1.1.

Supplementary figure 7.1 shows that nodes representing genes (Boolean variables) are constraint nodes (defined in Section 1) linked to reactions in HepatoNet1. The lookup tables specifying constraints as a function of the Boolean variable state contain identical reaction lists to the QSSPN model; the bounds for QSSPN node state of 0 and 2 were assigned to Boolean node states FALSE and TRUE. The objective function definitions are the same as used in QSSPN model. The full model definition is available in the model files included in software distribution accompanying this supplementary material; implementation of rFBA3 simulation is shown in supplementary figure 7.2.

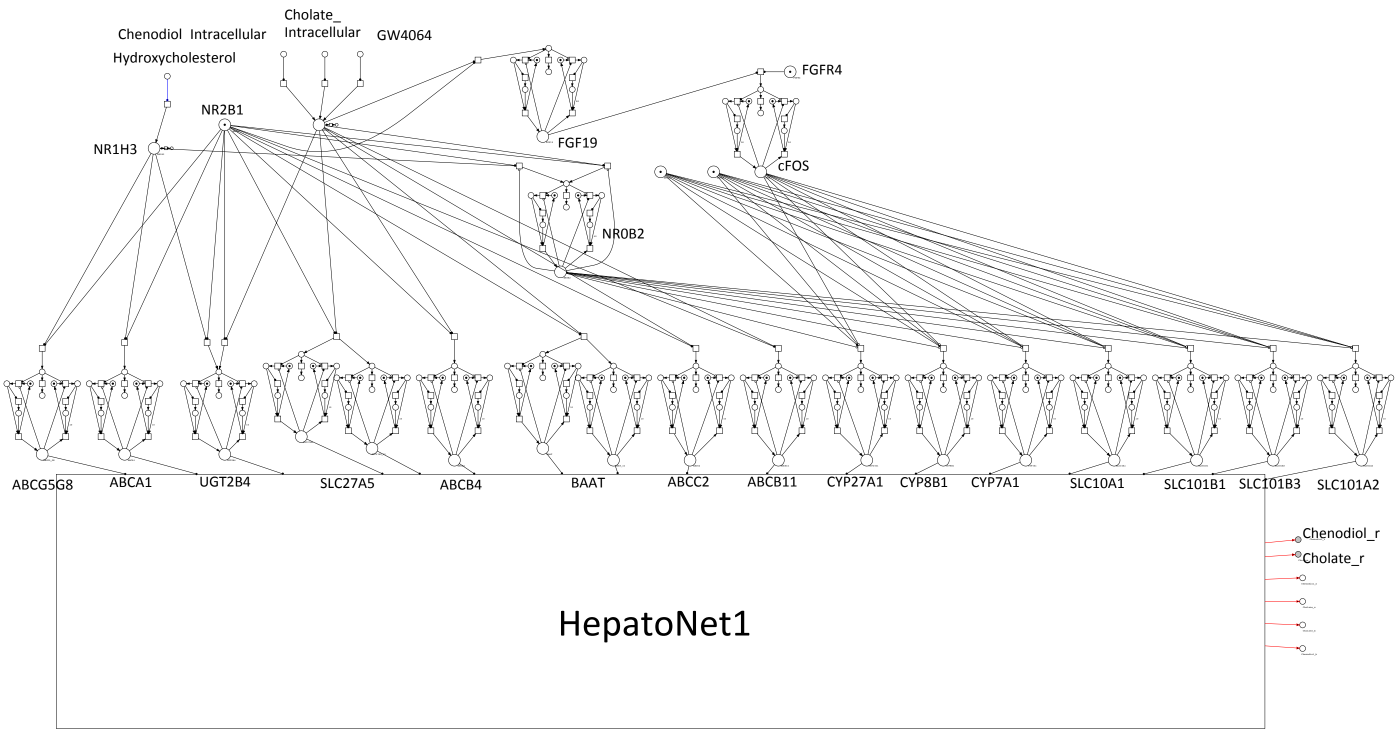

Supplementary Figure 7.2. rFBA3 model used for comparison simulations with QSSPN.

7.2.2 Results of rFBA simulations and comparison with QSSPN.

We have performed rFBA simulations of 14 benchmark experiments (Chapter 4.2) with three variants of the rFBA method. Perturbations to the model were implemented as follows; all simulations were run for 100 discrete timesteps, the simulation time and delays were selected to give best performance of rFBA methods against experimental datasets. We demonstrate that the major disadvantages of rFBA methodology, with respect to QSSPN, could not have been resolved by altering delays and simulation time.

The initial states for all simulations were set as follows. All genes, which were constitutively expressed ("TRUE" rule in Supplementary Table 7.1), were set to TRUE. All other genes were set to FALSE. Subsequently, simulations were run for 10 timesteps before any perturbation was applied. This allowed all Boolean variables to assume their state determined rules evaluated at an initial, unperturbed state.

Perturbations following initial unperturbed simulation period were implemented as follows. Cholesterol perturbation was set in exactly the same was as in QSSPN simulations; bounds of the FBA reaction modelling increased cholesterol variability were set to high values. GW4064 treatment was simulated by setting GW4064 variable to TRUE. FGF19 treatment was modelled by setting the state of FGF19 variable to TRUE. In simulations modelling SHP siRNA experiments, the NR0B2 gene was set to FALSE. Likewise anti–FGF19 antibody and anti-FGFR4 antibody experiments were simulated by setting respective gene variables to FALSE.

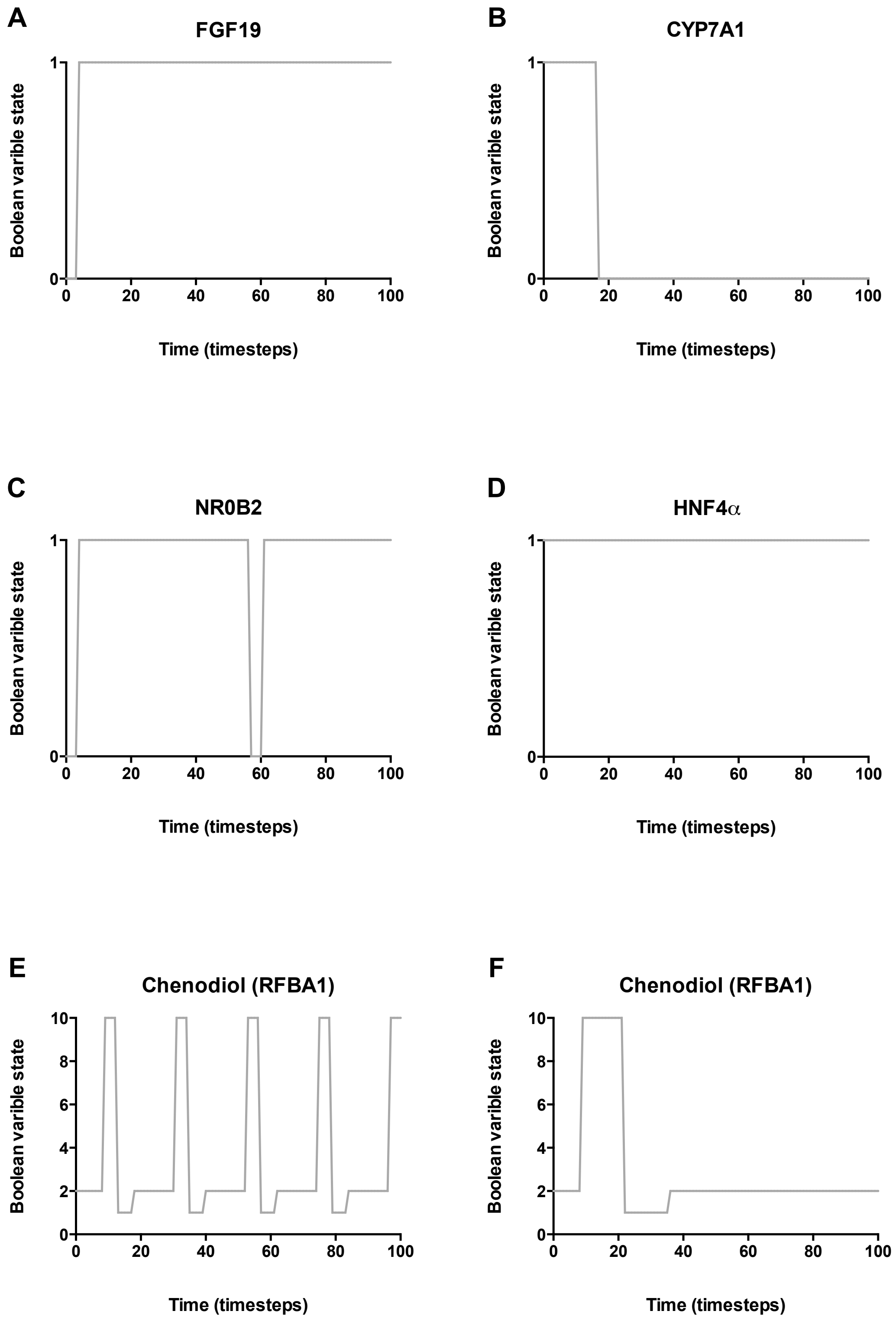

Supplementary Figure 7.3. Deterministic timecourses of selected rFBA simulations. A, B, C, D: Timecourses of Boolean variables representing FGF19, CYP7A1, NR0B2 and HNF4A genes, respectively, after GW4064 treatment. E, & F: Timecourses of Chenodiol bile acid objective function in RFBA1 simulation (without delays) and RFBA3 simulation (with gene specific activation and decay delays), respectively; objective function values are discretised to 10 integer levels using the same objective node lookup table as in QSSPN models.

Supplementary Figure 7.3 shows deterministic timecourses for selected rFBA simulations. Contrary to QSSPN, rFBA simulations produce single deterministic trajectories. Alternative sequences of events are not exploited. Two types of timecourses are generated: the trajectories of Boolean variables and trajectories of objective function. Boolean variable trajectories (Supplementary Figure 7.3 A-D) represent FALSE or TRUE variable states as 0 and 1, respectively. Alternatively, we can plot the values of FBA objective functions. Since in our implementation we use the same definition of objective functions as in QSSPN, we can plot the state of objective nodes to obtain timecourses directly comparable with those generated using QSSPN. The Boolean variable timecourses can be compared with experimental data and QSSPN results after being classified into dynamic behaviours. Definitions of activation, oscillation, burst and constant behaviours, defined in section 4.1, can be used to classify timecourses with two levels. Thus, trajectories shown in Supplementary Figure 7.3 A-D exhibit activation, inhibition, oscillation and constant behaviours, respectively. The objective function timecourses can also be classified into behaviours. The chenodiol bile acid objective exhibits oscillation behaviour in RFBA1 and burst behaviour in RFBA3 simulations, respectively. Assignment of increase, decrease and equal behaviours for rFBA simulations is described in the footnote of Supplementary Table 7.3 and discussed subsequent sections.

Supplementary Table 7.3. Comparison of QSSPN with rFBA.

Molecule	Experimental data	Experimental behaviour	rFBA1 behaviour	rFBA2 behaviour	rFBA3 behaviour	QSSPN behaviour
FGF19 transcript	Relative amount after treatment with GW4064 for 0, 0.6, 1, 3, 6, 24 hours (section 4.2.1).	activation	activation	activation	activation	activation
CYP7A1 transcript	Relative amount after treatment with GW4064 for 0, 0.6, 1, 3, 6, 24 hours (section 4.2.2).	inhibition	inhibition	burst	inhibition	inhibition
SHP transcript	Relative amount after treatment with GW4064 for 0, 0.6, 1, 3, 6, 24 hours (section 4.2.3).	burst	oscillation	oscillation	oscillation	burst
HNF4a transcript	Relative amount after treatment with GW4064 for 0, 0.6, 1, 3, 6, 24 hours (section 4.2.4).	constant	constant	constant	constant	constant
CYP7A1 transcript	Relative amount after treatment with FGF19 for 0, 0.6, 1, 3, 6, 24, 48 hours (section 4.2.5).	inhibition	inhibition	burst	inhibition	inhibition
SHP transcript	Relative amount after treatment with FGF19 for 0, 0.6, 1, 3, 6, 24, 48 hours (section 4.2.6).	burst	constant	constant	constant	burst
CYP7A1 transcript	Relative amount with respect to untreated control after treatment with FGF19 (section 4.2.7)	decrease	decrease*	decrease*	decrease*	decrease
CYP7A1 transcript	Relative amount with respect to untreated control after treatment with SHP siRNA (section 4.2.8)	increase	equal**	equal**	equal**	increase
CYP7A1 transcript	Relative amount with respect to untreated control after treatment with SHP siRNA and FGF19 (section 4.2.9)	decrease	decrease*	decrease*	decrease*	decrease
SHP transcript	Relative amount with respect to untreated control after treatment with FGF19 (section 4.2.10)	equal	equal**	equal**	equal**	equal
CYP7A1 transcript	Relative amount with respect to GW4064 treatment after FGF19 antibody treatment (section 4.2.11)	increase	undetermined***	undetermined***	undetermined***	equal
CYP7A1 transcript	Relative amount with respect to GW4064 treatment after FGFR4 siRNA (section 4.2.12)	increase	undetermined***	undetermined***	undetermined***	equal
Chenodiol	Physiological response to rise of cholesterol. (section 4.2.13)	burst	oscillation	burst	burst	burst
Cholate	Physiological response to rise of cholesterol. (section 4.2.13)	burst	oscillation	burst	burst	burst
**Number of correctly predicted experimental behaviours (TP)**			**7**	**7**	**9**	**12**
**Matthews Correlation Coefficient (MCC)**			**0.360**	**0.360**	**0.543**	**0.817**

* the gene exhibit constant level of 1 (TRUE) in control and inhibition behaviour in treated system.

** the rFBA trajectories exhibited the same behaviour in treated system and untreated control.

*** the gene exhibits oscillation behaviour in treated system and constant level of 1 (TRUE) in control. Thus behaviour cannot be classified to increase, decrease or equal.

Supplementary Table 7.3 shows direct comparison of QSSPN and rFBA simulations against 14 experimental datasets (sections1-13). **Results show that QSSPN achieves superior predictive power compared to any implementation of rFBA**. The QSSPN simulation correctly predicts 12/14 experimental behaviours, whereas the rFBA with variable delays (rFBA3) makes only 9/14 correct predictions. The rFBA with no delays (rFBA1) and (rFBA2) with constant delay, both predict correctly only 7/14 behaviours. Using MCC (section 4.3.1) as an error function, QSSPN outperforms all implemented rFBA approaches. QSSPN achieves an MCC of 0.817, while rFBA3, rFBA2 and rFBA1 only achieve MCCs of 0.543, 0.360 and 0.360, respectively.

The first important caveat of the rFBA approach, is the failure of rFBA1 method to reproduce homeostatic response of bile acid synthesis to a high cholesterol load. The timecourse of this simulation is shown in supplementary figure 7.3E. The trajectory exhibits oscillatory behaviour, and bile acid synthesis flux does not return to the basal level. The rFBA1 uses Boolean rules without delays; this is also the way in which rules are evaluated in COBRA toolbox. The correct physiological behaviour can be reproduced only if at least one class of gene activation/inactivation delays is introduced following original rFBA formulation (M. W. Covert *et al.*, 2008). This result shows that introduction of delays is vital for the quasi-steady state simulations that use Boolean rules to represent regulatory networks. This motivated our development of rFBA3, using a more general definition of delays than originally proposed by Covert and colleagues and so improving its predictive power.

All rFBA methods fail to predict burst behaviour of SHP transcript after GW4064 treatment (section 4.2.3). This behaviour is only correctly predicted by QSSPN. The timecourse of SHP transcript after GW4064 treatment obtained in rFBA3 simulation is shown on supplementary figure 7.3C, exhibiting oscillatory behaviour in contrast to experimental data and QSSPN which exhibit burst. This error occurs despite our attempts to account for high stability of SHP protein, accounted for in QSSPN (see main manuscript), to reproduce burst behaviour. In rFBA3 simulation we set a longer inactivation delay time for NR0B2 gene encoding SHP protein than that for other genes (50 timesteps for NR0B2, 10 timesteps for other genes). It is important to consider whether this error is a problem inherent to using rFBA gene regulatory networks, or whether we failed to identify proper parameter configuration. In particular, one may argue that if simulation time was set to 68 timesteps, the behaviour shown in supplementary figure 7.3C would be classified as burst. According to our results, the rFBA approach is not able to reproduce the major feature distinguishing burst from oscillation; the waiting time for the subsequent burst is much longer than the rise time of first burst. Although, we cannot guarantee that QSSPN simulations would not produce a second SHP peak for a much longer simulation time, the waiting time for second burst would be much longer than that for first burst. In rFBA simulations this is not possible to achieve even if gene specific activation and decay times are used. At the end of first oscillation the system returns to the state proceeding first peak and thus the second peak happens immediately afterwards. We cannot improve this situation by making the activation waiting time longer. This would simply result in the first peak appearing later and the second peak still appearing after a waiting time no longer than the first activation delay. We believe that the failure of rFBA methods to reproduce the burst behaviour in SHP transcript is general and cannot be resolved by adjustment of delay and simulation times.

The reason why rFBA failed to reproduce SHP transcript burst, while QSSPN was successful, is the fact that the rFBA gene regulatory network does not differentiate between transcription and translation processes. In QSSPN simulations, SHP transcript decays faster than SHP protein and stable protein inhibits transcript formation. Thus, fast transcript and protein production and fast mRNA decay is followed by a long period in which transcription is switched off by the presences of stable SHP protein. This leads to one peak of transcript activity, followed by a very long period in which the transcript is not expressed (burst behaviour). While, in principle, one could attempt to represent proteins and mRNAs as separate Boolean variables, this is beyond the scope of this comparison with previously published approaches. In all published work on rFBA, Boolean rules were used to implement gene regulatory network with gene activity as a whole represented by Boolean variables. We conclude that the QSSPN approach better predicts experimental observations for negatively autoregulated genes. This represents a significant advantage, as negative autoregulation is a common phenomenon in the molecular interaction networks of cells.

Results of sections 4.2.6-11 illustrate the advantages of QSSPN over rFBA in simulating experimental data on changes in relative molecular activity from *in vitro* experiments. In section 4.2.8 QSSPN is the only method that correctly predicts that relative CYP7A1 transcription is upregulated in SHP siRNA treated cells. In rFBA simulations, CYP7A1 exhibits the same activation behaviour in both treated and control system (equal behaviour). The QSSPN simulation shows that the number of trajectories exhibiting CYP7A1 activation is significantly higher in the SHP siRNA treated system than in an untreated control (Supplementary Figure 4.7). We could reproduce this effect only after assuming low basal expression rate of SHP gene. Even at basal levels, SHP can still activate with a low rate of 0.1, which is 1000 times lower than with full activation of the promoter. As a result, there is a degree of CYP7A1 repression in untreated controls, which is affected by siRNA treatment. The rFBA simulations did not reproduce these effects. As demonstrated in section 5, QSSPN parameters are qualitative and their choice does not require any more quantitative knowledge than setting of Boolean rules. In QSSPN the assumption of a basal expression rate or promoter leakage can be made without the knowledge of the quantitative values of transcription initiation rate.

Supplementary table 7.3 highlights further examples of difficulties in examining deterministic timecourses to analyse data on up/down regulation of genes in treated samples with respect to untreated control. For example, in rFBA simulations of experimental data shown in sections 11-12, the treated system exhibited oscillatory timecourse behaviour of CYP7A1 transcript, while the transcript was constantly activated in untreated controls. In this case it was not possible to decide whether transcript activity increased, decreased or did not change. We reflected this problem by introducing "undetermined" behaviour in the supplementary table 7.3. In QSSPN, the Monte Carlo sampling resulted in the numbers of trajectories exhibiting activation behaviour in both cases that could have been directly compared. Experiments where gene expression is compared in treated and untreated cells is common in molecular biology. Thus results sections 4.2.6-12 show that

QSSPN offers a distinct advantage over rFBA when comparing of simulation results with the plethora of qualitative experimental data available in the literature.

7.2 Conclusions.

The only previously published approach that can be applied to our test case study is rFBA (M. W. Covert *et al.*, 2001), where a Boolean gene regulatory network is integrated with FBA. However, it is still necessary to modify the original method for it to be applicable to the simulation of human cells. Other approaches cannot be adapted to our test case because they require quantitative parameters which are not available (Karr *et al.*, 2012), do not simulate gene regulation and signalling (Krauss *et al.*, 2012), do not perform dynamic simulations (Chandrasekaran and Price, 2010; Shlomi *et al.*, 2007) or model gene regulation and signalling alone, without including whole cell metabolism (Batt *et al.*, 2012; Ruths *et al.*, 2008).
QSSPN simulations achieved better predictive power than rFBA explained by the fundamental properties of both methods and not as a result of the QSSPN model better fitting our particular test case study. We show that our qualitative model of gene regulation, representing translation and transcription individually, provides better prediction of autoregulated gene dynamics than rFBA gene regulatory networks. We also demonstrate that Monte Carlo sampling of alternative event sequences is a more robust strategy than examination of a single deterministic trajectory when simulating changes of relative molecular activity.

Comparison between QSSPN and rFBA illustrates one more important feature of QSSPN: its flexibility in expressing different modelling assumptions. We show that PN can be used to implement synchronous Boolean gene regulatory network with delays and thus, the QSSPN method and algorithm can be used to build rFBA models. QSSPN gives users an unprecedented ability to apply different modelling assumptions to their system of interest and compare predictions with experimental results. As such, QSSPN represents a powerful tool for building models at a level of abstraction appropriate for the specific questions of researchers and available experimental data.

We conclude that from the modeller's perspective, QSSPN is the current method of choice for computer simulation of the molecular interaction network dynamics in human cells. Modellers can create large-scale models integrating existing whole-cell, steady-state metabolic models of human tissues and gene regulatory/signalling networks. These models can be analysed through dynamic simulation to investigate the full range of qualitative dynamic behaviours occurring in biological systems. Adoption of the Model Checking methodology, where simulation checks feasibility of qualitative dynamic behaviours exhibited by experimental data, facilitates direct comparison of simulation results with vast qualitative molecular biology knowledgebase. High-throughput transcriptomics and proteomics datasets typically show relative changes, and large scale reconstructions using the QSSPN framework will be directly comparable with these data. Extension of the regulatory part of the model to a larger scale, allowing incorporation of high-throughput data is an obvious future and computationally feasible (Section 8). We did not present a whole-cell scale signalling network simulation in this work because whole cell mechanistic model building is a long-term effort, requiring dedicated projects and is beyond the scope of this method validation.

We believe that QSSPN is an ideal approach for the iterative cycle of reconstruction, simulation and prediction and experimentation that will eventually lead to the prediction of genotype-phenotype relationships in human tissues, through mechanistic simulation of whole-cell molecular biology.

8 COMPUTATIONAL EFFICIENCY OF QSSPN

Here, we assess the computational efficiency of QSSPN, compare it with the computational costs of other approaches and argue that it is practical to use QSSPN for qualitative simulation of whole-cell molecular network models.

Calculation of the single example trajectory of QSSPN simulation for the WT version of our model took 3 minutes 18 seconds of CPU time on a single core Intel I7 2.6 GHz CPU. Therefore, collecting a sample of 120 trajectories, as used in all our calculations takes about 6 hours on a single core CPU. While this may seem a long time, it is important to note that Monte Carlo sampling is trivial to parallelize and that contemporary computers have multi-core CPUs. One hundred and twenty trajectories can be collected in 2 hours using 3 parallel processes run on a standard 4 core I7 processor. Our simulations of genotype-phenotype relationship were run on a 48 core Linux server, which let us complete calculations within approximately 2 hours. While, a 48 core computer is unlikely to feature on a researcher's desktop, it is an affordable proposition for a research group. Therefore, QSSPN is a practical method that can be applied by molecular biology research groups without the need for prohibitive investment into high performance computing hardware.

To assess the scalability of QSSPN to bigger models and compare its computational performance with rFBA let us first consider its performance bottleneck. The most computationally expensive step of QSSPN simulation is the execution of linear programming to evaluate objective function of the metabolic model. Evaluation of a single objective function (maximal flux towards Chenodiol) on HepatoNet1 model takes 0.189s. Our hepatocyte model contains 14 constraint nodes, each of them potentially requesting objective function evaluation with the whole-cell metabolic model. A key feature of the QSSPN algorithm that makes simulation practical is that objective functions are not re-evaluated if FBA reaction bounds have not changed since the last evaluation. This situation is quite frequent; for instance, firing of a transcription reaction, does not change state of the enzyme (constraint node) and so none of the FBA bounds changes. We would like to stress that this optimisation does not affect simulation results. If objective functions were re-evaluated at every simulation step, the software would simply be re-calculating exactly the same values. To quantify the benefit introduced by application of UpdateRequired variable, we set this variable TRUE for entire simulation, thus forcing calculation of all objective functions at every simulation step. Calculation of the single example trajectory of QSSPN simulation for the WT version of the model took 84 minutes 36 seconds of CPU time on a single core of Intel I7 2.6 GHz CPU. Thus, for our case study, algorithm optimisation resulted in an approximately 30-fold increase of performance without changing simulation results.

Assessing scalability of the QSSPN algorithm to models bigger than our test case is not straightforward. The number of objective function evaluations depends on the system and initial conditions. A system in which the regulatory network does not change any metabolic reaction bounds will be simulated very fast however, a system with complex dynamics will take much longer to evaluate. Dependence of simulation time on the system itself, rather than system size or a timestep is a common feature of Model Checking methods (Kwiatkowska *et al.*, 2011). Therefore, we ensured that our test case used full genome scale metabolic network model (hepatocyte) to ensure that the major computational cost is already tested on whole-cell scale model. While the regulatory network would be much larger in a whole-cell model, its evaluation does not represent a major computational cost. Calculation of propensity functions for thousands of dynamic transitions is still very fast compared to the Simplex algorithm applied to optimisation of a whole cell metabolic network. Moreover, we expect that in future whole-cell simulation of a particular biological experiment, the majority of dynamic transitions will be occurring in a regulatory sub-network activated to respond to particular experimental perturbation or a gene knockout. One of the basic properties of a cell is that its genetic material carries full genetic potential, but only a limited set of genes is expressed. To capitalise on this fundamental property of biological systems, we constructed our qualitative gene expression model in such a way that we change state of transcripts and proteins only if biologically meaningful change of regulatory conditions happen. This means, that we do not model constant levels of transcript or protein by explicitly simulating synthesis and degradation events that equate to a steady state. We execute synthesis and degradation transitions only if the regulatory event shifts the balance of synthesis and degradation, thus leading to a change in the steady state. In this way simulation of genes constitutively expressed under given conditions does not affect computational cost (See Section 3.1 of main manuscript). We, expect that in the whole-cell simulation the number of transitions, which are frequently executed and the number of timesteps in which evaluation of objective functions is requested will not necessarily be much larger than in the test case presented here. We model the complete regulatory network used by the cell to maintain homeostasis of essential products (primary bile acids) during physiological perturbation of increasing cholesterol load. We expect that whole cell models, like actual cells, will represent the whole genetic potential, but under particular simulation conditions will activate only a fraction of network connectivity. The whole-cell model will enable simulation of any relevant genetic and environmental perturbation, but a particular simulation addressing a particular research question is unlikely to be substantially more computationally demanding than the simulations presented here. Obviously, it is difficult for us to provide an exact estimate of the computational costs of large-scale models as these models would have to be reconstructed first. However, we are convinced that application of QSSPN in whole-cell mechanistic modelling is practical.

Since none of the existing implementations of quasi-steady state simulations integrating regulation and whole-cell metabolism are directly applicable to human cells, comparison of the computational performance of our software with these methods is very difficult. We would also like to stress that in none of the published studies where rFBA, iFBA, idFBA or "diverse mathematics" were used to simulate system dynamics, the authors use full GSMNs. The models used were either reduced metabolic networks or metabolic networks of a minimal genome. In the publication where full genome scale model of *E. coli* was used, regulatory rules were applied only to constraint steady states, rather than to perform dynamic rFBA simulation (M. W. Covert *et al.*, 2004). Therefore, our QSSPN simulations and our implementation of rFBA, is the first published case of integrating simulation of regulatory processes with full genome-scale FBA. The published versions of rFBA, iFBA and idFBA are likely to be faster than QSSPN, because only one objective function evaluation is performed per iteration. However, this is unacceptable for simulation of human cells as it implies reliance on biomass objective and simulation of cell culture

growth. Our implementation of rFBA is computationally more efficient than QSSPN. Simulation of rFBA timecourse for WT hepatocyte model takes 18 seconds on a single core of Intel I7 2.6 GHz CPU. The speed over QSSPN (3 minutes 18 seconds) results from coarse-graining of molecular interactions. The simulation trajectory involves far fewer iterations, since the state of a whole gene is changed at once, without updating transcripts, proteins and signalling networks separately. Since, simulation is deterministic only a single trajectory needs to be simulated. , While this simulation is more efficient than QSSPN, it has numerous disadvantages in terms of the predictive power, as previously discussed.

We conclude that from a computational point of view QSSPN is an expensive, but affordable method for qualitative, dynamic simulation of genome scale molecular interaction networks in human cells. Taking into account that it achieves better predictive power than the alternative and that the model allows representations of all levels of molecular organisation, we believe that it is the method of choice for simulation of human cell systems.

9 SOFTWARE AND MODEL DISTRIBUTION.

The model and simulation software are available in Supplementary file: `QSSPN_Supplement.zip` .

The open source QSSPN software is available under GNU GPL license. The ZIP archive contains the following files:

`bin/`	Directory containing executable software files.
`bin/LICENSE.txt`	The qsspn software is available under GNU GPL license. This is the license file.
`bin/qsspn-mac`	Command line executable binary file for MacOSX
`bin/qsspn-linux`	Command line executable binary file for Linux
`bin/spept2qsspn.py`	Python script converting models in Snoopy extended Petri Net format (*.spept) to QSSPN files (*.qsspn).
`src/`	Directory containing qsspn software source code.
`compile.sh`	Shell script that compiles qsspn software on MacOSX and Linux (tested with Fedora).
`clean.sh`	Shell script that cleans src directory from binaries created during compilation.
`HepatocyteQSSPN/`	The model of bile acid homeostasis in human hepatocyte.
`HepatocyteQSSPN/ HepatocyteQSSPN.spept`	The model file in Snoopy software format.
`HepatocyteQSSPN/ HepatoNet1.PIPES.sfba`	The SurreyFBA file with HepatoNet1 genome scale metabolic reaction network model.
`HepatocyteQSSPN/ HepatocyteQSSPN.pdf`	Snoopy Extended Petri Net file exported as PDF.
`HepatocyteQSSPN/ HepatocyteQSSPN.MAPK.pdf`	The sub-network of MAPK signalling exported as PDF.
`HepatocyteQSSPN/ HepatocyteQSSPN.sbml`	Snoopy Extended Petri Net file exported as SBML.
`HepatocyteQSSPN/ HepatocyteQSSPN.qsspn`	The model in QSSPN file format generated by `spept2qsspn.py` script.
`HepatocyteQSSPN/ HepatocyteQSSPN.ctrl.txt`	The QSSPN software control file.
`HepatocyteRFBA/`	The model of bile acid homeostasis in human hepatocyte constructed in RFBA framework. The delays are set for the best performing variant of the model (RFBA3). The *spept, *qsspn, *pdf , *ctrl.txt and *sbml files are provided.

9.1 Model files.

The `Hepatocyte` directory `QSSPN_Supplement` contains the QSSPN model of bile acid homeostasis in human hepatocytes. The following files are available.

The file `HepatocyteQSSPN.spept` can be viewed and edited with the general PN software Snoopy (http://www-dssz.informatik.tu-cottbus.de/DSSZ/Software/Snoopy). It shows connectivity of the Dynamic Transitions (Regulatory) part of the model. The QSSPN specific parameters are given in the comments sections of node, transition and edge symbols. The format of QSSPN parameter definitions is described in the QSSPN file format section below. One of the transitions, indicated by large rectangle, represents Quasi Steady State Fluxes (Metabolism) part of the model.

The QSSF part of the model is given in the file `HepatoNet1.PIPES.sfba`. This file contains HepatoNet1 model with boundary conditions set to the Physiological Input Physiological Output Set. The file is in SurreyFBA format file, which can be viewed and edited with text editors or JyMet GUI of SurreyFBA software (http://sysbio3.fhms.surrey.ac.uk). The file can be also used for Constrained Based Methods (CBM) simulations implemented in SurreyFBA.

The connectivity of the model can also be viewed in PDF format. The files `HepatocyteQSSPN.pdf` and `HepatocyteQSSPN.MAPK.pdf` have been created using EPS export from the Snoopy software and represent the network connectivity in vector graphics. The file `HepatocyteQSSPN.MAPK.pdf` shows MAPK signalling module, which is encapsulated within coarse transition "MAPK_Signalling".

We have used Snoopy to export our model in the SBML format. The `HepatocyteQSSPN.sbml` file represents the Dynamic Transitions part of the model. In the current version, QSSPN specific parameters are provided in annotation sections of SBML. The SBML representation of HepatoNet1 is available in original publications describing this model. Complete representation of QSSPN models in pure SBML is currently under development.

The `HepatocyteQSSPN.qsspn` file represents the model in the format of QSSPN command line software. This file has been created by `spept2qsspn.py` script available in `bin/` directory of supplementary material. The file can be read and modified with text editor. The format is documented below.

The file `HepatocyteQSSPN.ctrl.txt` contains simulation parameters, such as trajectory length, number of samples and output files. The file can be edited with text editor and its format is documented below.

9.2 QSSPN model file format.

The QSSPN file specifies the Dynamic Transition part of the model as well as constraint and objective nodes connecting Dynamic Transitions to Quasi Steady State Fluxes. First, the list of PN nodes is defined in the following format:

```
SUBSTANCES
  Name init   max type
  …
END
```

Each line between `SUBSTANCES` and END specifies one PN node. The fields have the following meaning:

`Name` – node name.
`init` – initial number of tokens associated with the node.
`max` – the maximal number of tokens.
`type` – node type, 1 – common node, 2 – objective node, 3 – constraint node.

Subsequently, the text between tags `REACTIONS` and `END` specifies PN transitions. Each transition is specified by the text between `INTERACTION` and `END` tags:

```
INTERACTION
   SUBSTRATE Name rate type delay
      ACTIVITY n
          t1  a1
          …
          tn  an
      END
   END
  PRODUCT Name
  CONSUMED Name
END
```

Each `SUBSTRATE` and `PRODUCT` tag specifies a PN pre-place or post-place, respectively. The `CONSUMED` tag specifies which of the pre-places change state upon firing of the transition. When transition fires it removes tokens from pre-places specified as `CONSUMED` and adds tokens to post-places. Thus, activators and inhibitors feature as transition pre-places, but are not specified as `CONSUMED`. The `ACTIVITY` section is associated with particular pre-place and provides a lookup table with contribution of the pre-place to transition propensity function defined in main manuscript. The variables have the following meaning:

`Name` – the name of PN node.
`rate` – transition rate.
`type` – transition type, SLOW (stochastic), INSTANT (immediate), FAST (continuous).
`delay` – Applies to SLOW (stochastic) transitions only. If delay is greater than 0 transition is treated as delayed with delay time set by this parameter.
`t, a` – Threshold and propensity function contribution. If the state of the pre-place node is greater or equal to `t` in particular row of the table and smaller than `t` in next row than the pre-place contribution to propensity function is set to `a`.

The text between `GSMN` and `END` describes connectivity between Dynamic Transitions (regulation) and Quasi Steady State Fluxes (metabolism). `MODEL` tag can be used to specify the path to “sfba” file with the QSSF model. Settings of `MODEL` tag are overridden by the value of `MODEL` tag in the control file. The `EXT_TAG` specifies externality tag in SurreyFBA file. This is the string at the end of metabolite name, which indicates external (unbalanced) metabolites in FBA model.

The objective node is specified by METABOLITE tag:

```
METABOLITE Name Objective
      ACTIVITY k
          t1  n1
          …
          tn  nk
      END
   END
```

where `Name` is the name of PN objective node and `Objective` is the name of flux or metabolite in the QSSF. The activity table maps real values of linear programming optimisation of QSSF objective to integer number of tokens.

The constraint node is specified by `ENZYME` tag:

```
ENZYME Name Flux
      ACTIVITY k
         n1          lb1     ub1
           …
         nk          lbk     ubk
      END
   END
```

where `Name` is the name of constraint node and Flux is the flux in the QSSF, which is constrained. The activity table maps an integer number of tokens n into lower (lb) and upper (ub) flux bound in QSSF.
Alternatively, the activity table can be applied to a list of fluxes rather than a single flux. This enables modelling of enzymes capable of catalysing multiple reactions in metabolic network. Instead of the flux name the keyword "list" is used. The list of m fluxes is provided between LIST and END tags. Each flux name is associated with weight w to further increase flexibility of expressing enzyme-reaction relationship. Each flux on the list is assigned bounds determined by activity table and multiplied by the weight.

```
ENZYME Name list
      ACTIVITY k
         n1          lb1     ub1
           …
         nk          lbk     ubk
      END
        LIST m
           Flux1 w1
         …
         Fluxm wm
      END
   END
```

9.3 QSSPN control file format

The `HepatocyteQSSPN.ctrl.txt` file can be edited with text editor. It specifies parameters of the simulation. The file has the following format:

```
MODEL path
NUMBER_OF_SAMPLES N
SEED seed
TIME_MAX tmax
MAXIMAL_TIMESTEP timestep

OUTPUT out
LOG log
MONITOR m

TARGET name t

PETRI_NET_MONITORS
name
…
END

HYBRID_SIMULATION3
```

The `path` specifies file containing metabolic network in SurreyFBA format. On some Linux distributions, the full path to the file may be required. The parameter `N` indicates the number of independent trajectories starting from the same initial conditions. The `seed` is the seed of random number generator. The `tmax` is the maximum simulation time. The trajectory stops after maximal time is reached. The maximal timestep parameter of the QSSPN algorithm is set to `timestep`.

The state of the system is recorded after each `m` iterations of QSSPN algorithm. Trajectory is written in tab separated text format to the file specified by out path. The names of PN nodes to be monitored in the trajectory file need to be listed between `PETRI_NET_MONITORS`

and END tags. Each name should be written in a separate line. The log file reports completion of each trajectory and can be used to monitor progress of the simulation. The log file also contains statistics of trajectory sampling.

The QSSPN trajectory samples are analysed to calculate the number of trajectories that exhibit certain behaviour. If a behaviour of interest can be expressed as a reachability problem (certain node reaching certain state) the TARGET tag can be used to facilitate calculations. In the log file the QSSPN software reports the number of trajectories, where target node specified by name reaches threshold of t. The trajectory is stopped after these conditions have been met. If the user wishes to calculate sample of trajectories which are not stopped before tmax and analyse them later, a dummy target that can never be reached needs to be specified (any PN node with threshold larger than its maximal number of tokens). For example in HepatocyteQSSPN.ctrl.txt we set:

```
TARGET  Chenodiol_r 100000
```

The HYBRID_SIMULATION3 indicates the function implementing QSSPN algorithm. Currently only this function is available, but in future versions we may implement functions performing more complex simulation protocols (e.g. node inactivation scans).

9.4 Running a simulation.

To convert model file from spept format of Snoopy Petri Net editor to qsspn format the following Unix command line needs to be executed in Hepatocyte/ directory of QSSPN_Supplement :

```
python ../bin/spept2qsspn.py HepatocyteQSSPN.spept 2 > HepatocyteQSSPN.qsspn
```

The spept2qsspn.py script is provided in the bin/ directory of Supplementary material. The HepatocyteQSSPN.spept is the original model file generated in Snoopy. The QSSPN file is printed to a standard output, which is re-directed to file.qsspn. The second command line parameter is an integer number specifying default maximal number of tokens of the PN node. The maximal number of tokens of the PN node is set to this number, unless specified otherwise by the MAX tag in the node comment section of the "spept" file.

The simulation on MacOSX operating system is run by the following command:

```
../bin/qsspn-mac HepatocyteQSSPN.qsspn HepatocyteQSSPN.ctrl.txt
```

The qsspn-mac binary is available in bin/ directory. To run simulation on Linux use qsspn-linux binary which is also available in bin/ directory. For other platforms, the source code in src/ directory needs to be compiled.

The files in Hepatocyte/ directory are set to simulate 1 example trajectory of simulation presented on Figure 3 of the main manuscript.

9.5 Simulation output file format.

Simulation produces tab separated tabular output containing sample of trajectories. The first field indicates sequential trajectory number in the sample. Second field is simulation time. The third field lists the name of stochastic transition that happened at that exact time. If no stochastic transition happened and only deterministic transitions were synchronously executed within the maximal timestep, the "none" keyword is given. The following fields give numbers of tokens associated with PN nodes specified in PETRI_NET_MONITORS section of the control file. The names of these nodes are given in the header line.

10 SUPPLEMENTARY REFERENCES

Baldazzi,V. *et al.* (2010) The Carbon Assimilation Network in Escherichia coli Is Densely Connected and Largely Sign-Determined by Directions of Metabolic Fluxes. *PLoS Comput. Biol.*, **6**, e1000812.
Baldi,P. *et al.* (2000) Assessing the accuracy of prediction algorithms for classification: an overview. *Bioinformatics*, **16**, 412–424.
Batt,G. *et al.* (2012) Genetic network analyzer: a tool for the qualitative modeling and simulation of bacterial regulatory networks. *Methods Mol Biol*, **804**, 439–462.
Bettenbrock,K. *et al.* (2006) A quantitative approach to catabolite repression in Escherichia coli. *J. Biol. Chem.*, **281**, 2578–2584.
Chandrasekaran,S. and Price,N.D. (2010) Probabilistic integrative modeling of genome-scale metabolic and regulatory networks in Escherichia coli and Mycobacterium tuberculosis. *Proc Natl Acad Sci U S A*, **107**, 17845–17850.
Chiang,J.Y.L. (2009) Bile acids: regulation of synthesis. *The Journal of Lipid Research*, **50**, 1955–1966.
Karr, JR. *et al.* (2012) A Whole-Cell Computational Model Predicts Phenotype from Genotype. *Cell*, **150**, 389–401.
Covert,M.W. *et al.* (2004) Integrating high-throughput and computational data elucidates bacterial networks. *Nature*, **429**, 92–96.
Covert,M.W. *et al.* (2008) Integrating metabolic, transcriptional regulatory and signal transduction models in Escherichia coli. *Bioinformatics*, **24**, 2044–2050.
Covert,M.W. *et al.* (2001) Regulation of Gene Expression in Flux Balance Models of Metabolism. *Journal of Theoretical Biology*, **213**, 73–88.
Gille,C. *et al.* (2010) HepatoNet1: a comprehensive metabolic reconstruction of the human hepatocyte for the analysis of liver physiology. *Molecular Systems Biology*, **6**, 1–13.
Guell,M. *et al.* (2009) Transcriptome Complexity in a Genome-Reduced Bacterium. *Science*, **326**, 1268–1271.
Krauss,M. *et al.* (2012) Integrating Cellular Metabolism into a Multiscale Whole-Body Model. *PLoS Comput. Biol.*, **8**, e1002750.
Kuhner,S. *et al.* (2009) Proteome Organization in a Genome-Reduced Bacterium. *Science*, **326**, 1235–1240.
Kwiatkowska,M. *et al.* (2011) PRISM 4.0: Verification of Probabilistic Real-time Systems. In, Gopalakrishnan,G. and Qadeer,S. (eds). Springer, pp. 585–591.
Lewis,N.E. *et al.* (2012) Constraining the metabolic genotype–phenotype relationship using a phylogeny of in silico methods. *Nature Publishing Group*, **10**, 291–305.
Li,T. and Chiang,J.Y.L. (2012) Bile Acid Signaling in Liver Metabolism and Diseases. *Journal of Lipids*, **2012**, 1–9.
Min Lee,J. *et al.* (2008) Dynamic Analysis of Integrated Signaling, Metabolic, and Regulatory Networks. *PLoS Comput. Biol.*, **4**, e1000086.
Ruths,D. *et al.* (2008) The Signaling Petri Net-Based Simulator: A Non-Parametric Strategy for Characterizing the Dynamics of Cell-Specific Signaling Networks. *PLoS Comput. Biol.*, **4**, e1000005.
Schellenberger,J. *et al.* (2011) Quantitative prediction of cellular metabolism with constraint-based models: the COBRA Toolbox v2.0. *Nat Protoc*, **6**, 1290–1307.
Shlomi,T. *et al.* (2007) A genome-scale computational study of the interplay between transcriptional regulation and metabolism. *Molecular Systems Biology*, **3**.
Song,K.-H. *et al.* (2008) Bile acids activate fibroblast growth factor 19 signaling in human hepatocytes to inhibit cholesterol 7α-hydroxylase gene expression. *Hepatology*, **49**, 297–305.
Steggles,L.J. *et al.* (2007) Qualitatively modelling and analysing genetic regulatory networks: a Petri net approach. *Bioinformatics*, **23**, 336–343.
Varma,A. and Palsson,B.O. (1994) Stoichiometric flux balance models quantitatively predict growth and metabolic by-product secretion in wild-type Escherichia coli W3110. *Appl Environ Microbiol*, **60**, 3724–3731.
Yus,E. *et al.* (2009) Impact of Genome Reduction on Bacterial Metabolism and Its Regulation. *Science*, **326**, 1263–1268.
